# Grape Flavonoid Evolution and Composition Under Altered Light and Temperature Conditions in Cabernet Sauvignon (Vitis vinifera L.)

**DOI:** 10.3389/fpls.2019.01062

**Published:** 2019-11-08

**Authors:** Erna H. Blancquaert, Anita Oberholster, Jorge M. Ricardo-da-Silva, Alain J. Deloire

**Affiliations:** ^1^Department of Viticulture and Oenology, Stellenbosch University, Matieland, South Africa; ^2^Department of Viticulture and Enology, University of California, Davis, Davis, CA, United States; ^3^LEAF-Linking Landscape, Environment, Agriculture and Food - Instituto Superior de Agronomia, Universidade de Lisboa, Lisboa,Portugal; ^4^SupAgro, Department of Biology and Ecology, Montpellier University, Montpellier, France

**Keywords:** Cabernet Sauvignon, tannin, anthocyanins, flavonols, light quality, light quantity, PAR, temperature

## Abstract

The evolution of flavonoids under altered temperature and light conditions in the fruit zone was followed in Cabernet Sauvignon (*Vitis vinifera* L.) grapes during ripening. The study was conducted over two consecutive seasons in 2010/2011 and 2011/2012 comprising two main treatments in which the light quantity was manipulated in the bunch zone: (1) standard (STD) with no lateral shoot or leaf removal and (2) leaf removal west (LRW) treatment with leaf removal on the western side of the bunch zone. Furthermore, the light quality was altered by installing ultraviolet B-suppression sheets within the bunch zone in both seasons. Tannin evolution was dependent on the prevailing light quality/quantity and temperatures during berry development in a particular season. Grape seed tannin accumulation coincided with seed development and commenced at the early stages of berry development. Seed proanthocyanidin composition was not influenced by the treatments. The largest impact on proanthocyanidin accumulation and structure in the skin was due to seasonal variations highlighting the complex interaction between light quality and/or quantity across the two growing seasons and eventually the complex interaction with temperature. Flavonol accumulation was significantly influenced by the light quality, which is known to be the main abiotic driver of flavonol biosynthesis regulation. Anthocyanin concentration and content were largely dependent on the temperature and light quality in a particular season. Anthocyanin composition was altered by the season rather than the treatment.

## Introduction

Cabernet Sauvignon (*Vitis vinifera* L. cv.) is one of the most planted red grape cultivars globally, which is also true for the Stellenbosch Wine of Origin District in South Africa South African Wine Industry Statistics (SAWIS, 2016). Despite the importance of this variety for winemaking, there are still important research questions that should be addressed. One such question is about the impact of light quality and quantity in interaction with temperature on berry flavonoid evolution/biosynthesis and concentration at the microclimatic/bunch level.

Flavonoids perform major roles in plants such as pollen fertilization, auxin transport regulation, pigmentation, defense against pathogens and pests, and protection from ultraviolet (UV) radiation (Ribereau-Gayon and Glories, 1986; Santos-Buelga and Scalbert, 2000; Winkel-Shirley, 2001; Adams, 2006). Additionally, flavonoids are important in wine because of their contribution to color, taste, mouthfeel, and the potential beneficial role in human health (Ribereau-Gayon and Glories, 1986; Santos-Buelga and Scalbert, 2000; Winkel-Shirley, 2001; Adams, 2006). The three main groups of flavonoids identified in red grape berries are flavan-3-ols (tannin), anthocyanin, and flavonols.

Flavan-3-ols include a range of polyphenolic compounds that include flavan-3-ol monomers, dimers, and various oligomers and polymers that are connected by interflavan linkages (C4–C8 or C4–C6) called condensed tannins or proanthocyanidins (Adams, 2006). Proanthocyanidins are the most abundant class of grape phenols in the grape berry and are present in the seeds, skins, pulp, and stems (Jordão et al., 2001; Adams, 2006; Cerpa-Calderón and Kennedy, 2008). Flavonols are colorless compounds that accumulate after flowering and during ripening (Flint et al., 1985; Cheynier and Rigaud, 1986; Price et al., 1995; Downey et al., 2003; Mattivi et al., 2006). They contribute to wine color by forming co-pigment complexes with anthocyanins (Scheffeldt and Hradzina, 1978; Boulton, 2001). Moreover, flavonols are UV protectants and act as free radical scavengers (Flint et al., 1985). Research also found that flavonols participate in plant–pathogen interactions (Macheix et al., 2005; Adams, 2006). Quercetin-3-*O*-glucoside and quercetin-3-*O*-glucuronide have been identified as the main flavonols within the grape berries (Cheynier and Rigaud, 1986; Price et al., 1995; Downey et al., 2003; Mattivi et al., 2006). Anthocyanins are the pigmented compounds responsible for the color of red grapes and wine (Mattivi et al., 2006). Anthocyanins are synthesized and accumulate from véraison in the berry skin of most grapes (Jordão et al., 2001; Guan et al., 2012). However, some *Vitis vinifera* cultivars (i.e., Alicante Bouschet) and non-*Vitis vinifera* (i.e., hybrid cultivars) contain anthocyanins also in the pulp and are known as *teinturier* cultivars (Spayd et al., 2002).

A number of factors have been identified that can influence flavonoid accumulation and composition in grapes. This includes abiotic factors such as light, temperature, and water status as well as cultivar, crop level, nutritional status, soil type, and plant growth regulators (Asen et al., 1972; Scheffeldt and Hrazdina, 1978; Flint et al., 1985; Crippen and Morrison, 1986; Ricardo-da-Silva et al., 1991; Ricardo-da-Silva et al., 1992a; Ricardo-da-Silva et al., 1992b; Gao and Cahoon, 1994; Price et al., 1995; Dokoozlian and Kliewer, 1996; Smith and Markham, 1998; Mazza et al., 1999; Haselgrove et al., 2000; Bergqvist et al., 2001; Boulton, 2001; Jordão et al., 2001; Kennedy et al., 2002; Ojeda et al., 2002; Downey et al., 2003; Kolb et al., 2003; Ryan and Revilla, 2003; Downey et al., 2004; Mori et al., 2005; Cortell and Kennedy, 2006; Downey et al., 2006; Mori et al., 2007; Ristic et al., 2007; Cerpa-Calderón and Kennedy, 2008; Koyama and Goto-Yamamoto, 2008; Berli et al., 2011; Lorrain et al., 2011; Azuma et al., 2012; Cohen et al., 2012; Gregan et al., 2012; Guan et al., 2012). The main flavan-3-ol subunits present in grape seeds are (+)-catechin, (−)-epicatechin and (−)-epicatechin-3-*O*-gallate. The main terminal subunit is (+)-catechin, and the main extension subunit is (−)-epicatechin (Romeyer et al., 1986; Prieur et al., 1994; Downey et al., 2004). Grape skins differ from seeds because (−)-epigallocatechin and a lower proportion of galloylated units are present in the skins (Escribano-Bailón et al., 1995; Souquet et al., 1996; De Freitas et al., 2000; Kennedy et al., 2001; Downey et al., 2003; Obreque-Slier et al., 2010). Furthermore, a higher degree of polymerization occurs in grape skins (Adams, 2006). In berry skin, (+)-catechin has been identified as the main terminal and extension subunit skins (Escribano-Bailón et al., 1995; Souquet et al., 1996; De Freitas et al., 2000; Kennedy et al., 2001; Downey et al., 2003; Obreque-Slier et al., 2010). The accumulation of flavonoids and their genes is up-regulated with exposure to light, while shading down-regulates the gene expression (Ryan and Revilla, 2003; Mori et al., 2007). Flavonol amounts increased in grapes exposed to high levels of sunlight (Crippen and Morrison, 1986; Price et al., 1995; Haselgrove et al., 2000; Azuma et al., 2012; Martínez-Lüscher, 2019). Varying results have been obtained on the effect of light exposure on anthocyanin accumulation. Gao and Cahoon (1994) and Dokoozlian and Kliewer (1996) reported a reduction in the anthocyanin content when fruits were shaded without altering the temperatures. Other authors reported no change in the anthocyanin content of shaded fruit compared with exposed fruit (Price et al., 1995).

The potential effects of light (quality vs UV-B and quantity) and temperature (interaction or separately) on phenolic grapevine berry biosynthesis, structure, and concentration are still not clear, and further research is needed. The aim of this study was to investigate the potential effect/role of light quality and quantity (while the temperature was monitored) on berry flavonoid accumulation and composition in Cabernet Sauvignon (*Vitis vinifera* L.) by manipulating the light in the fruit zone over two seasons. This work is part of a larger study in which grapes were harvested sequentially to investigate the link between fruit and wine chemical composition and wine sensory profiles (Blancquaert, 2015; Blancquaert et al., 2019).

## Materials and Methods

### Vineyard Characteristics

The study was conducted during two growing seasons (2010/2011 and 2011/2012) in a Stellenbosch University vineyard (GPS coordinates: 33°56′42″S 18°27′43″E). The vineyard is planted with *Vitis vinifera* L. cv. Cabernet Sauvignon clone CS 388C, grafted onto 101–14 Mgt (*Vitis riparia* × *Vitis rupestris*). The row orientation was north–west/south–east. The vines are trained on a six-wire vertical trellis system. Additionally, the block was subjected to irrigation during critical phenological stages (e.g., fruit set and véraison) and as required throughout the season. This was done to have a predawn leaf water potential between 0 and −0.3 MPa (Deloire and Heyns, 2011). The study comprised two main treatments with altered bunch microclimates in both seasons: no lateral shoot or leaf removal in the bunch zone (STD, shaded bunches) and leaf removal on the western side of the bunch zone (leaf removal west (LRW), exposed bunches in the afternoon sun) (**Table 1**; **Table S1**). All the leaves were removed just after berry set corresponding to growth stage 27 (Eichhorn and Lorenz system) on the western side of the canopy at the fruit zone level (± 35–40 cm above the cordon) (Coombe, 1995).

**Table 1:** Treatment description for 2010/2011 and 2011/2012 seasons.

Treatments	
2010–2011	2011–2012
No lateral shoots or leaves were removed in the bunch zone, and no water shoots were suckered **Shaded (control) (STD)**	
Leaf removal west side of the bunch zone just after berry set **Exposed—Leaf removal west (LRW)**	
Control treatment and UV sheet (Perspex^®^ Opal 050) on the western side of the bunch **STD with decreased UV-B radiation** **(STD-UV-B)**	Leaf removal on both sides of the canopy (in the bunch zone) and (Perspex^®^ Opal 050) on both sides of the bunch zone **Leaf removal with decrease in PAR and UV-B radiation and 2xOp50 UV sheets added on both sides of the bunch zone** **LR (-UV-B,-PAR) (shaded without leaves and laterals)**
Leaf removal west and UV sheet (Perspex^®^ Opal 050) on the western side of the bunch **LRW with decreased UV-B radiation** **(LRW-UV-B)**	Leaf removal on both sides of the canopy (in the bunch zone), and UV sheet (UHI) extruded clear acrylic was used on both sides of the bunch zone **Leaf removal with decreased UV-B radiation and 2xUHI UV sheets added on both sides of the bunch zone** **LR(-UV-B, 2xUHI)**

Furthermore, to study the effect of UV light on fruit growth and composition, supplementary treatments were applied. A UV sheet (Perspex^®^ Opal 050, Perspex South Africa Pty Ltd, Umbogintwini) (**Table S1**), reducing the UV-B radiation, was added to the control/STD (STD-UV-B) and leaf removal west (LRW-UV-B) treatment in 2010/2011. After analyzing the results in 2010/2011, it became apparent that the row orientation is north–west/south–east contrary to previous belief of a north–south orientation. This resulted in treatments receiving longer exposure in the morning (eastern side of the canopy) and shorter exposure in the afternoon side (western side of the canopy). Therefore, UV-B-suppression sheets were installed on both sides of the canopy after the leaves were removed during the 2011/2012 season. In addition to the Perspex^®^ Opal 050 sheets (used in 2010/2011), a clear acrylic UV sheet (extruded high impact (UHI)) was used during the 2011/2012 season. The latter resulted in the following treatments: LR (-UV-B, -PAR) shaded without leaves and laterals, and LR (-UV-B, 2xUHI) (**Table 1**). These sheets were installed just after fruit set at ±35 cm above the cordon and suspended on 1.2-m custom-made poles with hinges to open for sampling and the spraying program. The treatments were applied in a randomized block design. Each treatment was carried out in five replicates, and each replicate comprised three panels (18 vines per replicate).

### Temperature Measurements

Microclimate within the canopy and bunch zone was determined within each treatment with a Tinytag (Tinytag Plus TGP-1500, West Sussex, UK). The Tinytags were placed on the surface of the berry skins for the respective measurements. Berry temperatures were measured every 15 min from December until March (96 measurements daily) in both seasons. The thermocouples were attached on the outside of the berry surface. The average berry temperature was calculated per hour throughout the season for each treatment. Thermal time in degree days (DD, °C) was calculated on each day and summed throughout each season. The latter was computed, as follows:

$$DD=\frac{1}{n}\sum_{i=0}^{n}(T-Tb),$$

where *n* is the number of averaged data logger readings per day, *T* is the daily mean temperature (°C), and *T*_b_ is the base temperature (10°C) for grapevine growth.

### Light Measurements

The incidence of photosynthetically active radiation (PAR) in microeinsteins per square meter per second was determined with a ceptometer (LP-80 AccuPAR, Decagon Devices Inc. Nebraska, USA). Five measurements were taken in the middle of the bunch zone on clear, sunny days at 11:00, 13:00, and 15:00 on 3 days during the growing season in both seasons.

### Sampling Procedure and Preparation for Analyses

Sampling occurred at regular intervals throughout the season (**Table S2**). Sampling was conducted between 06:00 and 08:00 at each sampling date from after fruit set until harvest: 13–116 days after anthesis (DAA) during the 2010/2011 season and 26–130 DAA in the 2011/2012 season (**Table S2**). Sampling corresponded with the Eichhorn and Lorenz (E-L) system and started at stage 29 (pea size) until stage 38 (harvest) for phenolic analyses (Coombe, 1995). Two berries from 10 bunches were sampled from each of the five treatment replicates in the middle of the bunch and kept separate for phenolic analyses. The berries of the LRW treatment were only sampled from the exposed bunches.

### Chemicals

All chromatographic solvents were of high-performance liquid chromatography (HPLC) grade. Ethanol, acetone, acetonitrile, methanol (≥99.9%), l-ascorbic acid, gallic acid, (+)-catechin, (−)-epicatechin, and quercetin were obtained from Sigma-Aldrich (Johannesburg, South Africa). Quercetin-3-glucoside was obtained from Fluka (Buchs, Switzerland), malvidin-3-glucoside from Polyphenols Laboratories AS (Norway), and acetic acid and orthophosphoric acid from Riedel-de Haën (Seelze, Germany). Purified water was obtained from a Milli-Q filtration system (Millipore Filter Corp., Bedford, MA, USA).

### Extraction of Grape Seeds and Skins

The berries were processed immediately after sampling for the phenolic analysis. Twenty berries from each treatment replicate were weighed. The berries were then frozen in liquid nitrogen. The berry samples were manually separated into skins and seeds, rinsed with water, and dried with tissue paper. Isolated skins and seeds were weighed. The extraction was performed as described by Panprivech et al. (2015). The aqueous solution was frozen overnight, lyophilized, and stored at −20°C until further analysis was performed. The dried tannin extract representing one biological repetition of 20 berries was re-dissolved in 10 mL of methanol for seeds and 5 mL of methanol for skins before reversed-phase high-performance liquid chromatography (RP-HPLC) analysis.

### Analysis of Phenolic Compounds by RP-HPLC

Monomeric and polymeric procyanidins (seed), proanthocyanidins (skin) (tannins), flavonols, and anthocyanins were quantified using RP-HPLC based on a method by Peng et al. (2001, 2002). The dried seed and skin extracts were re-dissolved and filtered using a 0.45-μm Millipore filter (Millipore, Bedford, Mass, USA) before injection and placed in a 1.5-mL amber HPLC vial. Tannin elutes as an unresolved peak at the end of the run. This method gives an estimation of tannin content and can show trends among samples. A Hewlett Packard Agilent 1260 series HPLC system equipped with a diode array detector (Agilent Technologies, Palo Alto, CA, USA) was used. Separations were carried out on a polystyrene/divinylbenzene reversed-phase column (PLRP-S, 100Ǻ, 250 × 4.6 mm, 5 µm) protected by a guard cartridge with the same packing material (PLRP-S, 10 × 4.6 mm). All materials were purchased from Polymer Laboratories (Shropshire, UK). All the conditions were the same as the method previously published (Peng et al., 2002).

Phenols were quantified using external calibration standards: (+)-catechin hydrate, (−)-epicatechin, gallic acid, malvidin-3-*O*-glucoside, and quercetin-3-*O*-glucoside (Sigma-Aldrich, Johannesburg, South Africa). Monomeric and dimeric flavan-3-ols and polymeric phenols were quantified at 280 nm as mg/L catechin units with a quantification limit of 0.78 mg/L and epicatechin with a limit of quantification (LOQ) of 0.89 mg/L. Gallic acid was quantified at 280 nm in gallic acid units with a LOQ of 0.05 mg/L. The corresponding limit of detection (LOD) for the monomeric and dimeric flavan-3-ols and polymeric phenols was 0.23 mg/L for (+)-catechin units and 0.26 mg/L for (−)-epicatechin. Flavonol glycosides were quantified at 360 nm as mg/L quercetin-3-*O*-glucoside with a LOQ of 0.20 mg/L. The following flavonols were identified: (i) quercetin-3-*O*-rutinoside, (ii) quercetin-3-*O*-galactoside, (iii) quercetin-3-*O*-glucoside, and (iv) quercetin-3-*O*-glucuronide. Anthocyanins and polymeric pigments were quantified at 520 nm as mg/L malvidin-3-*O*-glucoside with a quantification limit of 0.19 mg/L.

### Condensed Tannin Analysis by Acid-Catalyzed Cleavage in the Presence of Phloroglucinol

Compositional analysis of proanthocyanidins was carried out following acid-catalyzed cleavage in the presence of excess phloroglucinol (phloroglucinolysis) as described in Oberholster et al. (2013). The method provided information regarding the subunit composition, mean degree of polymerization (mDP), percentage of galloylation (%G), and the percentage of prodelphinidin units (%P) in grape seeds and skins, where applicable. The proanthocyanidin cleavage products were determined by RP-HPLC. The conditions for the chromatographic separation are described in Oberholster et al. (2013).

### Statistics

All analyses were carried out using Statistica 12 (Statsoft Inc., Tulsa, USA). Mixed-model repeated-measures (2010/2011 = 8; 2011/2012 = 10) analyses of variance (ANOVAs) were used and Fisher’s least significant difference (LSD) corrections were used for *post hoc* analyses. Significant differences were judged on a 95% significance level (*p* ≤ 0.05). Pearson’s correlation coefficients were used to construct heatmap.2 R function using R software (R Foundation for Statistical Computing, Vienna, Austria). The distribution of grape flavonoid datasets was analyzed with principal component analysis (PCA) using R software (R Foundation for Statistical Computing, Vienna, Austria).

## Results and Discussion

### Growing Degree Days

The accumulation of growing degree-days (GDD) was determined from December until March in both seasons. The amount of GDD for the 2010/2011 season was 1,262 and for the 2011/2012 season 1,451 (base temperature of 10°C). The accumulation of fruit thermal degree-days (DD, microclimate) was affected by the season and by the treatments (**Table 2**). The DD among the treatments in the 2011/2012 season was lower than that in the 2010/2011 season. The pattern of growing degree accumulation varied among the two seasons, as the macroclimate in the 2010/2011 season was characterized by continuous drought and heat throughout the summer (VinPro, 2011). On the other hand, the 2011/2012 season was, however, considered as an ideal growing season with a cool, and lengthened, harvesting period without rain or prolonged heat (VinPro, 2012). As anticipated, the STD treatment had the lowest DD, and LRW had the highest accumulated DD in the 2010/2011 season. The addition of the UV-B-suppression sheets altered the fruit temperatures in the STD-UV-B treatment as it had a higher DD than the STD (**Table 2**). This suggests that the leaf layers in the STD-UV-B treatment in combination with the UV-B sheet retained the heat and resulted in increased DD due to amplified solar radiation in the fruit zone. In the 2011/2012 season, the fruit from the LR (-UV-B, -PAR) (shaded without leaves and laterals) and LR (-UV-B, 2xUHI) treatments had the lowest and highest DD, respectively (**Table 2**). These results can be ascribed to the spectral properties of the sheets that were used in the study. The Perspex Opal 50 sheet (Op50) has a shading coefficient of 0.47, which resulted in more shading of the fruit, when compared with the UHI acrylic sheet with a shading coefficient of 1.0 (**Table S1**). This result agrees with that of Spayd et al. (2002), who observed that clusters exposed to UV-radiation-reducing sheets had higher DD values than exposed fruit.

**Table 2:** Accumulated thermal time and berry temperature and the average number of hours at thresholds in 2010/2011 and 2011/2012 seasons.

Season	Thermal time (DD)^a^	Berry temp (°C)		Number of hours berry temperature within the indicated temperature range (per day)				
		Mean	Max	<20	20–25	25–30	30–35	>35
**2010–2011** **Treatments**								
STD	731.3	23.4	32.5 b	9.3 b	5.5 a	4.5 a	3.8 b	0.9 d
LRW	757.8	23.9	35.4 a	9.4 a	5.2 b	3.5 c	4 b	2.0 a
STD-UV-B	756.1	23.8	33.8 b	9.5 b	4.8 c	3.7 c	4.5 a	1.6 b
LRW-UV-B	746.3	23.6	33.7 b	9.3 b	5.5 a	4 b	3.9 b	1.4 c
***p*-value**		ns	***	***	***	***	**	***
**2011–2012** **Treatments**								
STD	684.6	23.8 ab	40.4 a	10.4 c	4 b	3.3 c	3.2 b	3.1 b
LRW	686.7	23.2 ab	37.1 b	10.5 b	4.1 a	3.6 b	3.3 b	2.4 c
LR (-UV-B, -PAR)	680.9	22.8 b	34.5 c	10.7 a	4 ab	3.9 a	3.5 ab	1.8 d
LR (-UV-B, 2xUHI)	729.7	24.2 a	39.6 a	10.5 bc	3.5 c	2.5 d	3.7 a	3.8 a
***p*-value**		*	***	***	***	***	*	***

^a^Thermal time in degree days over the season. STD (shaded/control); LRW (leaf removal west); STD-UV-B (STD with decreased UV-B radiation; LRW-UV-B (LRW with decreased UV-B radiation); LR (-UV-B, -PAR) (leaf removal with decreased UV-B radiation and 2xOp50 UV sheets added on both sides of the bunch zone); LR (-UV-B, 2xUHI) (leaf removal with decreased UV-B radiation and 2xUHI UV sheets added on both sides of the bunch zone). Different letters indicate significant differences at p ≤ 0.05, 0.01, and 0.001, respectively; ns, not significant.

Significant differences (*p* ≤ 0.001) in the maximum temperatures at the different phenological stages were observed in both 2010/2011 and 2011/2012 seasons (**Table S3**). Smart (2010) suggested that fruit hot spots in compact bunches with low winds may occur within a season, which might have been the case in 2011/2012 for STD having higher mean maximum temperatures. Further analysis of the minimum, mean, and maximum temperatures at each phenological stage shows this phenomenon persisted within this season (data not shown). Smart and Sinclair (1976) reported that temperature is directly associated with the incident radiation. It is therefore difficult to separate the effects of light and temperature during fruit development. Berries in the LRW and, to a lesser extent, LRW-UV-B treatments received more direct sunlight as they were more exposed and therefore subjected to heating of the surface of the berry, while the STD treatments (STD, STD-UV-B) had indirect sunlight. In the 2011/2012 season the PAR and percentage light intensity in the shaded treatments (STD and LR (-UV-B, 2xOp50) were significantly lower (*p* ≤ 0.001) than the LRW and LR (-UV-B, 2xUHI) treatments (**Table 3**).

**Table 3:** Photosynthetically active radiation (PAR) and percentage light in the bunch zone in the 2010/2011 and 2011/2012 seasons.

2010–2011			2011–2012		
Treatments	PAR^a^	% light	Treatments	PAR^a^	% light
STD	175.3 bc	0.10 c	STD	72.0 b	0.06 c
LRW	517.7 a	0.29 a	LRW	278.9 a	0.18 ab
STD-UV-B	115.3 c	0.06 c	LR (-UV-B, -PAR)	98.4 b	0.07 cb
LRW-UV-B	260.2 b	0.16 b	LR (-UV-B, 2xUHI)	424.4 a	0.19 a
***p*-value**	***	***	***p*-value**	***	***

^a^Photosynthetically active radiation (μE·m^−2^·s^−1^). STD (shaded/control); LRW (leaf removal west); STD-UV-B (STD with decreased UV-B radiation; LRW-UV-B (LRW with decreased UV-B radiation); LR (-UV-B, -PAR) (leaf removal with decreased UV-B radiation and 2xOp50 UV sheets added on both sides of the bunch zone); LR (-UV-B, 2xUHI) (leaf removal with decreased UV-B radiation and 2xUHI UV sheets added on both sides of the bunch zone). Different letters indicate significant differences at p ≤ 0.05, 0.01, and 0.001, respectively; ns, not significant.

### Berry Growth and Development

The pattern of berry growth followed a typical double sigmoid pattern in both seasons. However, there was no significant difference in the average berry weight among the four treatments in the 2010/2011 season, while a significant difference (*p* ≤ 0.001) in the average berry weight was observed among the four treatments in 2011/2012 (**Table 4**).

**Table 4:** Mean berry weights (*n* = 3) determined during the 2010/2011 and 2011/2012 seasons.

2010–2011		2011–2012	
Treatments	Berry weight	Treatments	Berry weight (g)
STD	0.9	STD	1.0 a
LRW	0.8	LRW	0.9 b
STD-UV-B	0.9	LR (-UV-B, -PAR)	0.9 b
LRW-UV-B	0.9	LR (-UV-B, 2xUHI)	0.8 c
***p*-value**	ns	***p*-value**	***

STD (shaded/control); LRW (leaf removal west); STD with decreased UV-B radiation (STD-UV-B); LRW with decreased UV-B radiation (LRW-UV-B); LR (-UV-B, -PAR) (leaf removal with decreased UV-B radiation and 2xOp50 UV sheets added on both sides of the bunch zone); LR (-UV-B, 2xUHI) (leaf removal with decreased UV-B radiation and 2xUHI UV sheets added on both sides of the bunch zone). Significance Different letters indicate differences at p ≤ 0.05, 0.01, and 0.001, respectively; ns, not significant.

Mean berry weight of berries grown under lower light intensities (STD, LRW-UV-B, and STD-UV-B) was slightly higher than that of the berries grown under higher light intensities (LRW) in 2010/2011. A similar pattern was observed in the 2011/2012 season (**Table 4**). This phenomenon could be due to the shaded berries having a lower transpiration rate, which influenced the turgor pressure, resulting in enlargement of the berry as previously reported by other authors (Crippen and Morrison, 1986; Reynolds et al., 1986; Price et al., 1995). The berries from the LR (-UV-B, 2xUHI) treatment had considerable lower berry weight than do the other three treatments, which can be ascribed to higher exposer to solar radiation and lower shading ability, which resulted in higher transpiration rate.

**Figure 1:**
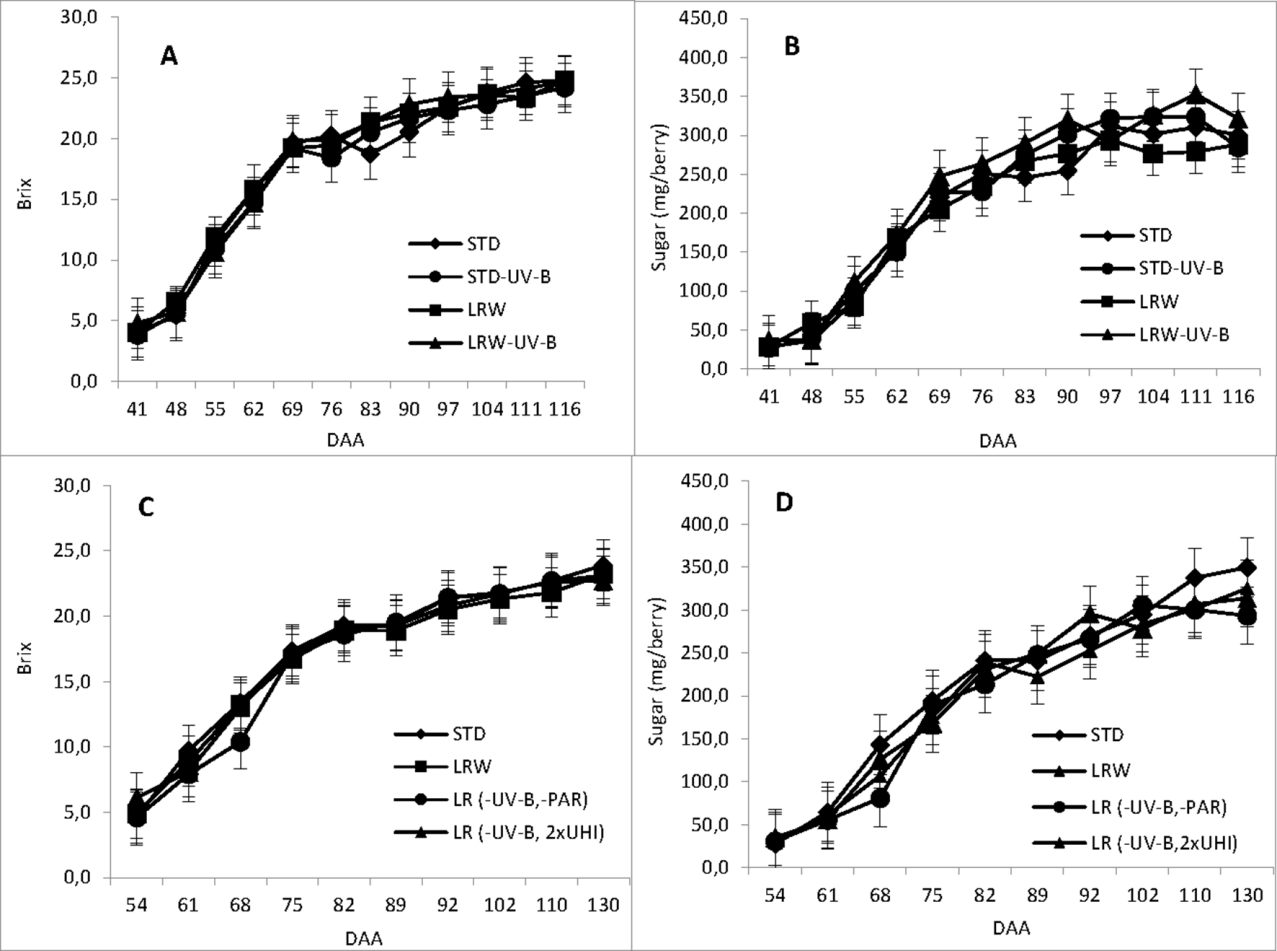
Total soluble solids and sugar accumulation determined in days after anthesis (DAA). **(A)** 2010/2011 TSS accumulation, **(B)** 2010/2011 sugar accumulation, **(C)** 2011/2012 TSS accumulation and **(D)** 2011/2012 sugar accumulaiton in 2011/2012. mEach value represents the mean of 3 replicates ± standard error (Blancquaert, 2015).

**Figure 1** and **Table S4** show the accumulation of total soluble solids and sugar (mg/berry) during the ripening period and the mean values in the 2010/2011 and 2011/2012 season for each of the respective treatments. An active period of sugar accumulation was noted per berry from véraison at 41 DAA and 54 DAA in the respective seasons. Sugar accumulation for all treatments followed a similar trend for both seasons, slowing down around 102 DAA in both seasons. Our findings support the findings of Buttrose et al. (1971) who reported similar effects of air temperature on berry size and total soluble solids that varied with the duration of exposure on berry growth stage.

The mean sugar content (mg/berry) differed significantly among treatments in both the 2010/2011 (*p* ≤ 0.001) and 2011/2012 (ꛝ*pင* ≤ 0.05) seasons. The mean sugar content in the 2010/2011 season was the highest in the LRW-UV-B treatment (231 mg/berry). The mean sugar content in the remaining treatments was 208.5, 204.8, and 216.8 mg/berry for STD, LRW, and STD-UV-B, respectively. In the 2011/2012 season, STD treatment had the highest sugar content (216.5 mg/berry), while LR (-UV-B, 2xUHI), LRW, and LR (-UV-B, 2xOp50) exhibited sugar contents of 205.8, 199.6, and 198.3 mg/berry, respectively. The differences in the sugar content can be attributed to the variation in the berry size, which was altered by the exposure at a given temperature and light intensity. Our findings support a study by Buttrose et al. (1971) that reported similar effects of air temperature on berry size and total soluble solids that varied with the duration of exposure on berry growth stage.

### Evolution of Grape Seed Procyanidins and Proanthocyanidins During Ripening

The main seed flavan-3-ol monomers identified were (+)-catechin, (−)-epicatechin, and (−)-epicatechin-3-*O*-gallate (data not shown). This is in agreement with the findings of several authors (Kennedy et al., 2000a; Kennedy et al., 2000b) who found that (+)-catechin, (−)-epicatechin, and (−)-epicatechin-3-*O*-gallate were the main seed flavan-3-ol monomers in different grape cultivars. Procyanidin B2 (EC-(4β-8)-Ec) was the most abundant dimer in the seeds of the two measured: B1 (EC-(4β-8)-Cat) and B2 (data not shown). Our results are in agreement with other authors (Ricardo-da-Silva et al., 1991; Ricardo-da-Silva et al., 1992a; Ricardo-da-Silva et al., 1992b; Prieur et al., 1994; De Freitas et al., 2000) who reported that B2 was the predominant dimer in grape seeds irrespective of the grape cultivar.

**Figure 2:**
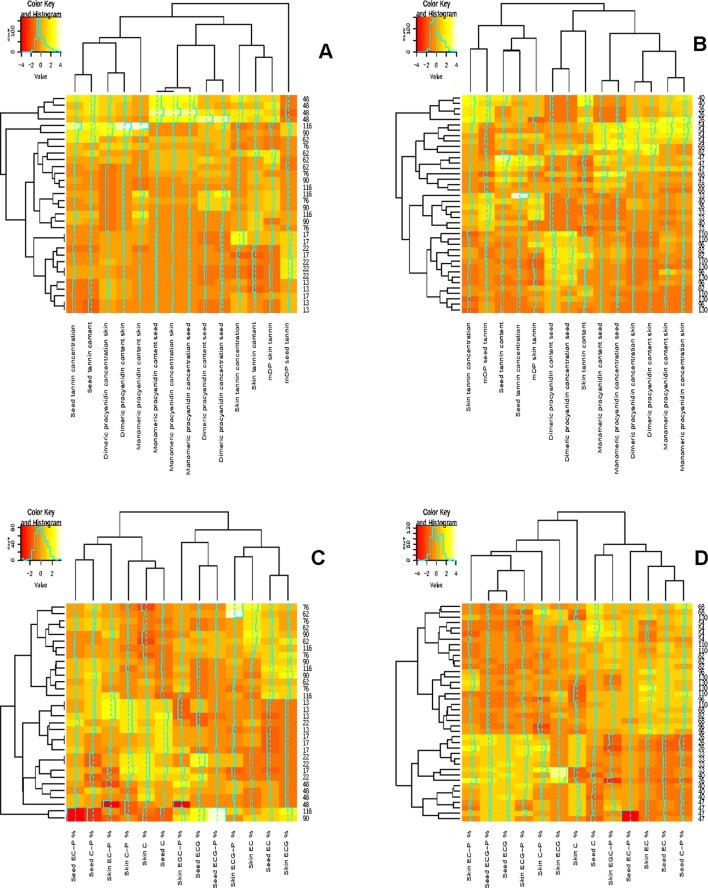
Correlation analysis showing clustered image maps of the correlation between grape seed and skin composition during ripening. **(A)** 2010-2011 grape seed and skin flavan-3-ol evolution during ripening **(B)** 2011-2012 grape seed and skin flavan-3-ol evolution during ripening, **(C)** 2010-2011 grape seed and skin compoition during ripening and **(D)** 2011-2012 grape seed and skin composition during ripening.

The concentration and content of individual monomers and dimers followed a similar pattern increasing from fruit set (13-22 DAA in 2010/2011 and 36-40 DAA in 2011/2012) (**Figures 2A, B**). A maximum was reached close to véraison (48 DAA in 2010/2011 and 54–68 DAA in 2011/2012) followed by a decrease until harvest (116 DAA in 2010/2011 and 130 DAA in 2011/2012) in both seasons (**Figures 2A, B**). The decrease in the monomer and dimer concentrations and contents confirms the findings of other studies. These studies reported that monomer and dimer syntheses occur before véraison, followed by a decrease that can be ascribed to a reduction in the extractability thereof (Kennedy et al., 2000a; Kennedy et al., 2000b).

When evaluating STD and LRW, the two treatments that were consistent between the two seasons, the mean monomer and dimer concentrations and contents of STD treatment, were similar between the seasons (**Table 5**). These results indicate minimal light and temperature effects, which are consistent with the findings of Dokoozlian and Kliewer (1996) and Downey et al. (2004), who suggested that shading resulted in minimal variation in seed chemistry. The mean monomer and dimer concentrations of the LRW treatment were also similar between the two seasons (**Table 5**). In the treatments with the UV-B-suppression sheets, we can see that the exclusion of UV-B radiation had an impact on the overall monomer and dimer concentrations and contents, but it was not consistent.

**Table 5:** The seasonal mean monomer/dimer and seed and skin tannin concentration and content in 2010/2011 and 2011/2012.

Treatment	Monomer and dimer seed concentrations (mg/g seed)	Monomer and dimer seed contents (mg/berry)	Total seed tannin concentration (mg/g seed)	Total seed tannin content (mg/berry)	Monomer and dimer skin concentrations (mg/g skin)	Monomer and dimer skin contents (mg/berry)	Total skin tannin concentration (mg/g skin)	Total skin tannin content (mg/berry)
**2010/2011**								
**Standard (control)**	5.89	0.33	44.61 a	2.20	0.093 a	6.67 a	0.010 a	0.668 a
**Leaf removal west**	4.93	0.26	43.26 a	2.02	0.065 b	7.06 a	0.006 b	0.693 a
**STD-UV-B**	4.88	0.27	39.96 ab	1.96	0.039 c	6.54 a	0.003 c	0.634 a
**LRW-UV-B**	5.37	0.28	37.58 b	1.75	0.065 b	4.78 b	0.006 b	0.448 b
**Significance**	ns	ns	*	ns	***	*	***	*
**2011/2012**								
**Standard (control)**	5.41 a	0.30 a	44.88 a	2.40	0.090 ab	5.591	0.010 a	0.509 b
**Leaf removal west**	0.34 c	0.29 a	42.22 a	2.36	0.116 a	6.372	0.010 a	0.588 a
**LR (-UV-B, 2xOp50)**	0.09 c	0.01 b	42.80 a	2.31	0.086 b	6.117	0.007 ab	0.566 ab
**LR(-UV-B, 2xUHI)**	2.94 b	0.25 a	25.88 b	2.24	0.068 b	6.237	0.005 b	0.533 ab
**Significance**	***	***	***	ns	*	ns	*	*

Each value represents the mean of five replicates at eight sampling dates in 2010/2011 and five replicates at 10 sampling dates in 2011/2012. Means in columns followed by a different letter are significantly different within one season. Monomers: C, (+)-catechin; EC, (−)-epicatechin; ECG, (−)-epicatechin-3-O-gallate. Dimers: B1, Ec-(4β-8-)Cat; B2, Ec-(4β-8-Ec. Treatments: STD (shaded/control); LRW (leaf removal west); STD with decreased UV-B radiation (STD-UV-B); LRW with decreased UV-B radiation (LRW-UV-B); LR (-UV-B, -PAR) (leaf removal with decreased UV-B radiation and 2xOp50 UV sheets added on both sides of the bunch zone); LR (-UV-B, 2xUHI) (leaf removal with decreased UV-B radiation and 2xUHI UV sheets added on both sides of the bunch zone). Different letters indicate significant differences at p ≤ 0.05, 0.01, and 0.001, respectively; ns, not significant.

The pattern of seed tannin concentration (mg/g seed) and content (mg/berry) differed, according to RP-HPLC determination, between the investigated seasons (**Figure 2**). The 2010/2011 season was characterized by an increase in the seed tannin concentration and content until véraison (48 DAA), followed by a decrease and another increase from 76 DAA in all the treatments until harvest (**Figures 2A, B**). In the 2011/2012, tannin accumulation increased until véraison or 2 weeks prior to véraison and then fluctuated except for treatment UHI (**Figure 2B**). Our results are in agreement with the findings of other authors who reported that the genes responsible for seed tannin biosynthesis are switched on after fertilization (Dixon et al., 2005). A maximum seed tannin concentration is reached close to véraison in Monastrell, Cabernet Sauvignon, and Shiraz, and then their concentration or their extractability decreases towards harvest (Kennedy et al., 2000a; Kennedy et al., 2000b; Bogs et al., 2005; Cortell and Kennedy, 2006; Downey et al., 2006). It has been reported that temperature and light conditions play an essential role in berry development. Dokoozlian (2000) reported that germination and pollen growth are greatly reduced or even inhibited when temperatures fall below 15.6° or exceed 37.8°C. From our study, it appeared that the seed development after flowering could potentially be influenced by prevailing temperatures. In the 2010/2011 season, December 2010 (berry set) was characterized by warm and dry periods, which could have contributed to favorable conditions for seed development, which resulted in a higher seed number and therefore influencing the evolution of the seed flavan-3-ol monomers, dimers, and total tannin. In the 2011/2012 season, mid-October to November of 2011 (flowering until berry set) had temperatures considered as below normal with November being 2°C colder than average, which resulted in a long, prolonged flowering period. However, the treatments and the temperature loggers were only installed after flowering, and it is difficult to assess the impact of temperature at flowering, which will have a direct impact on the seed number and size per berry in a particular season. We hypothesize that the evolution of the flavan-3-ol monomers, dimers, and total tannin were a function of the seed number per berry and were not influenced by the light and temperature conditions during the growing season.

### Evolution of Grape Skin Procyanidins and Proanthocyanidins During Ripening

The main flavan-3-ol monomers identified in the skins were (+)-catechin, (−)-epicatechin, and (−)-epicatechin-gallate; the dimers were EC-(4β-8)-Cat (B1) and EC-(4β-8)-EC (B2) in both seasons (data not shown). Many authors do not analyze skin flavan-3-ol monomer concentrations due to the complex interactions among sugars and other phenolics resulting in low concentrations of flavan-3-ol monomers during quantification (Monagas et al., 2003; Cortell and Kennedy, 2006). The accumulation pattern of skin monomers and dimers differed among the two seasons (**Figures 2A, B**). In general, the shaded STD treatment had the highest flavan-3-ol monomer and dimer concentrations and contents in 2010–2011 (**Figure 2A**). However, the other shaded treatment (STD-UV-B) had the lowest concentration and content. In the latter, UV-B radiation was additionally influenced, indicating the potential impact of UV-B radiation on flavan-3-ol synthesis. Downey et al. (2004) also found an increase in the monomer concentration and content in shaded canopies and a decrease in exposed fruit.

Differences in the concentration and content of total skin tannins were observed between the two seasons among all the treatments (**Table 5**). Overall, the skin tannin content was higher in the 2010/2011 season when compared with the 2011/2012 season (**Table 5**). Our results show that skin tannin reaches a maximum at véraison followed by a decrease, similar to the findings of other authors (Cortell et al., 2005; Hanlin and Downey, 2009). Differences between the sample preparation and analytical method used in our study could be a potential contributor to the variation in the evolution of the skin tannin concentration and content. The RP-HPLC quantification of tannin concentration and content is an underestimation of the tannin as some is lost on the baseline (Peng et al., 2001; Peng et al., 2002). This study indicates that light quantity and quality have a potential impact on flavan-3-ol and tannin accumulation in the skin. These findings also highlight the photo-protective role tannin play in the berry skin, which is supported by Downey et al. (2004) and Pastor del Rio and Kennedy (2006).

### Grape Seed Compositional Changes During Ripening

Seed tannin compositional data by phloroglucinolysis revealed that the terminal seed flavan-3-ol subunits were (+)-catechin, (−)-epicatechin, and (−)-epicatechin-3-*O*-gallate (**Figures 2C, D**; **Tables S5** and **S6**). The proportional composition of terminal subunits changed throughout berry development in both seasons (**Tables S5** and **S6**). The compositional changes in the terminal subunits during berry development were also observed by other authors (Kennedy et al., 2000a; Kennedy et al., 2000b; Downey et al., 2003). The seasonal impact on the seed proanthocyanidin terminal subunit composition was larger than the treatment impact. This is primarily due to the higher light intensities observed in the 2010/2011 season and lower light intensities in the 2011/2012 season.

(−)-Epicatechin was the main constituent of the seed extension subunits with (+)-catechin and (−)-epicatechin-3-*O*-gallate being present in lower proportions in both seasons (**Tables S5** and **S6**), similar to findings of other authors (Prieur et al., 1994; Cortell et al., 2005; Pastor del Rio and Kennedy, 2006). The proportional composition of extension subunits changed throughout berry development in both seasons (**Tables S5** and **S6**). The proportional compositional changes during ripening correspond with those of other studies for different cultivars (Kennedy et al., 2000b; Peng et al., 2001; Downey et al., 2003; Obreque-Slier et al., 2010). Our results agree with that of Fujita et al. (2007) and Cohen et al. (2008) who reported minimal variation in the seed proanthocyanidin composition with shading, heating, and cooling of berries.

Seed mDP varied between 2.7 and 8.8 in the 2010/2011 season and 2.9 and 7.7 in the 2011/2012 season during berry development among treatments (**Tables S5** and **S6**). Our findings are within the range found by other authors (Downey et al., 2003, Monagas et al., 2003, Chira et al., 2009). Similar to Obreque-Slier et al. (2010), the seed mDP increased from harvest to overripe stage. In the 2011/2012 season, the respective treatments had higher amounts of extension subunits at the beginning of berry ripening than the 2010/2011 season, resulting in higher mDP (**Tables S5** and **S6**). The decrease of mDP and avMM from fruit set corresponds with the findings of other authors (Reynolds et al., 1986; Price et al., 1995; Downey et al., 2003; Cortell et al., 2005; Chira et al., 2009).

The percentage of galloylated derivatives was determined during both seasons (**Table 6**; **Tables S5** and **S6**) with similar values to those reported by Chira et al. (2009). A significantly higher (*p* ≤ 0.001) percentage of galloylation was observed in the STD treatment when compared with the other three treatments in 2010/2011 (**Table 6**). During the 2011/2012 season, no significant differences were observed between the galloylation percentages among the treatments, suggesting that galloylation was influenced more by the season than the applied treatments (**Table 6**).

**Table 6:** Mean seed tannin structural characteristics in 2010/2011 and 2011/2012 seasons.

2010/2011				2011/2012			
Treatment	Seed % G	mDP	avMM	Treatment	Seed % G	mDP	avMM
**Standard (control)**	5.5 ± 5.1 a	6.1 ± 2.4 a	1,804.1 ± 736.4 a	**Standard (control)**	7.8 ± 7.0	5.0 ± 1.8 b	1,518.6 ± 590.5 cb
**Leaf removal west**	2.7 ± 1.5 b	5.4 ± 1.9 b	1,582.5 ± 555.8 b	**Leaf removal west**	7.5 ± 6.6	5.2 ± 1.7 a	1,581.6 ± 569.8 ab
**STD-UV-B**	0.6 ± 0.5 d	4.5 ± 1.5 c	1,309.0 ± 449.3 c	**LR (-UV-B, -PAR)**	7.6 ± 7.4	4.8 ± 1.7 b	1,456.4 ± 565.9 c
**LRW-UV-B**	1.6 ± 1.4 c	5.1 ± 2.3 b	1,499.2 ± 673.9 b	**LR (-UV-B, 2xUHI)**	7.9 ± 6.5	5.3 ± 1.6 a	1,587.9 ± 544.9 a
***Significance***	***	***	***	***Significance***	ns	***	***

Means in columns followed by a different letter are significantly different within one season. Mass conversion based on percent recovery of proanthocyanidin by phloroglucinolysis; %G, percentage galloylation; mDP, mean degree of polymerization; avMM, average molecular mass. STD (shaded/control); LRW (leaf removal west); STD with decreased UV-B radiation (STD-UV-B); LRW with decreased UV-B radiation (LRW-UV-B); LR (-UV-B, -PAR) (leaf removal with decreased UV-B radiation and 2xOp50 UV sheets added on both sides of the bunch zone); LR (-UV-B, 2xUHI) (leaf removal with decreased UV-B radiation and 2xUHI UV sheets added on both sides of the bunch zone). Different letters indicate significant differences at p ≤ 0.05, 0.01, and 0.001, respectively; ns, not significant.

### Grape Skin Compositional Changes During Ripening

(+)-Catechin, (−)-epicatechin, and (−)-epicatechin-3-*O*-gallate were identified as the grape skin proanthocyanidin terminal subunits (**Table 7**; **Tables S7** and **S8**). (+)-Catechin was the predominant compositional contributor, with epicatechin and (−)-epicatechin-3-*O*-gallate present in lower proportions or not detected (**Tables S7** and **S8**). This is in agreement with the findings of several other authors (Souquet et al., 1996; Kennedy et al., 2001; Downey et al., 2003; Monagas et al., 2003; Cortell and Kennedy, 2006; Ristic et al., 2007). There was a significant difference (*p* ≤ 0.001) in the mean (+)-catechin and (−)-epicatechin-3-*O*-gallate terminal subunit contribution between the two seasons (**Table 7**).

**Table 7:** Proportions of mean grape skin terminal subunits in 2010/2011 and 2011/2012 seasons. Means in columns followed by a different letter are significantly different within one season.

2010/2011				2011/2012			
Treatment	C	EC	ECG	Treatment	C	EC	ECG
**Standard (control)**	79.2 ± 17.7 b	9.9 ± 9.3 b	10.4 ± 11.7 a	**Standard (control)**	83.9 ± 11.7 a	15.5 ± 12.7	3.1 ± 3.1 b
**Leaf removal west**	79.6 ± 18.3 b	14.6 ± 13.1 a	5.8 ± 6.9 b	**Leaf removal west**	81.7 ± 12.7 ab	15.3 ± 10.9	3.1 ± 3.9 b
**STD-UV-B**	84.8 ± 13.6 a	8.8 ± 10.0 b	6.4 ± 6.9 b	**LR (-UV-B, -PAR)**	61.2 ± 1 2.2 c	15.6 ± 13.7	21.6 ± 13.2 a
**LRW-UV-B**	86.4 ± 12.8 a	3.9 ± 7.6 c	8.7 ± 9.5 a	**LR (-UV-B, 2xUHI)**	79.8 ± 15.2 b	17.0 ± 14.1	3.2 ± 2.3 b
***Significance***	***	***	***	***Significance***	***	ns	***

Percent composition of proanthocyanidin terminal skin subunits C, (+)-catechin; EC, (−)-epicatechin; ECG, (−)-epicatechin-3-O-gallate. STD (shaded/control); LRW (leaf removal west); STD with decreased UV-B radiation (STD-UV-B); LRW with decreased UV-B radiation (LRW-UV-B); LR (-UV-B, -PAR) (leaf removal with decreased UV-B radiation and 2xOp50 UV sheets added on both sides of the bunch zone); LR-UV-B, 2xUHI (leaf removal with decreased UV-B radiation and 2xUHI UV sheets added on both sides of the bunch zone). Different letters indicate significant differences at p ≤ 0.05, 0.01, and 0.001, respectively; ns, not significant.

(−)-Epigallocatechin was the predominant extension subunits followed by epicatechin in grape skins. Lower levels of (+)-catechin and (−)-epicatechin-3-*O*-gallate were found in both seasons (**Table 8**; **Table S7** and **S8**). These results are in agreement with other studies that found similar extension subunit proportions in skins (Kennedy et al., 2001; Downey et al., 2003; Hanlin and Downey, 2009). Our results contradict those of Fernandez et al. (2007), who identified epicatechin as the main contributor to skin extension subunits with lower (−)-epigallocatechin proportions present when studying Carménère skins. However, the (+)-catechin and (−)-epicatechin-3-*O*-gallate proportions obtained during both seasons were similar to those of Fernandez et al. (2007). In our study, light exposure did not have a significant impact on the extension unit composition in either of the seasons investigated (**Figures 2C, D**).

**Table 8:** Proportions of mean grape skin proanthocyanidin extension subunit in 2010/2011 and 2011/2012 seasons. Means in columns followed by a different letter are significantly different within one season.

2010/2011					2011/2012				
Treatment	C	EC	ECG	EGC	Treatment	C	EC	ECG	EGC
**Standard (control)**	1.9 ± 0.5 b	41.2 ± 2.9	0.3 ± 0.2 ab	56.6 ± 3.1	**Standard (control)**	1.2 ± 0.1 b	39.3 ± 2.4 ab	0.7 ± 0.3 b	58.8 ± 2.6
**Leaf removal west**	2.1 ± 0.5 a	40.1 ± 4.0	0.4 ± 0.5 a	57.4 ± 4.1	**Leaf removal west**	1.3 ± 0.1 b	38.8 ± 2.3 b	0.7 ± 0.3 b	59.2 ± 2.6
**STD-UV-B**	1.9 ± 0.3 ab	40.6 ± 2.5	0.2 ± 0.1 b	55.0 ± 2.8	**LR (-UV-B, -PAR)**	1.3 ± 0.2 a	39.4 ± 2.8 ab	0.6 ± 0.4 c	58.8 ± 4.7
**LRW-UV-B**	2.0 ± 0.3 b	42.2 ± 6.5	0.2 ± 0.1 b	55.5 ± 8.3	**LR (-UV-B, 2xUHI)**	1.2 ± 0.17 b	39.7 ± 2.3 a	0.8 ± 0.4 a	58.3 ± 2.6
***Significance***	**	ns	***	ns	***Significance***	***	*	***	ns

Percent composition of proanthocyanidin extension skin subunits C, (+)-catechin; EC, (−)-epicatechin; ECG, (−)-epicatechin-3-O-gallate; EGC, (−)-epigallocatechin. STD (shaded/control); LRW (leaf removal west); STD with decreased UV-B radiation (STD-UV-B); LRW with decreased UV-B radiation (LRW-UV-B); LR (-UV-B, -PAR) (leaf removal with decreased UV-B radiation and 2xOp50 UV sheets added on both sides of the bunch zone); LR-UV-B, 2xUHI (leaf removal with decreased UV-B radiation and 2xUHI UV sheets added on both sides of the bunch zone). Different letters indicate significant differences at p ≤ 0.05, 0.01, and 0.001, respectively; ns, not significant.

In the 2011/2012 season, the lowest average polymer length was observed in the LR (-UV-B, 2xUHI) treatment, while the LR (-UV-B,-PAR) (shaded without leaves and laterals) treatment showed higher mDPs (**Table 9**). The low mDP observed in the LR (-UV-B, 2xUHI) treatment can be a result of the high PAR (**Table 3**) that may have been above optimal levels. However, this is not supported by LRW treatment that had similar high PAR values combined with higher temperatures in 2010/2011. Chorti et al. (2010) reported that excessive sunlight exposure could result in excessive sunburn, which could influence skin proanthocyanidins in the grape berry. Additionally, Lacampagne et al. (2010) suggested that skin mDP is correlated with the state of skin cell walls. Skin tannins exhibit a higher degree of polymerization than seed tannins and are expressed as the mDP (Adams, 2006). Where similar mDP values were obtained between the seeds in the respective seasons, skin mDPs were higher in 2011/2012 when compared with 2010/2011 (**Table 9**).

**Table 9:** Mean skin tannin structural characteristics in 2010/2011 and 2011/2012 seasons.

2010/2011					2011/2012				
Treatment	Skin % G	Skin % P	mDP	avMM	Treatment	Skin % G	Skin % P	mDP	avMM
**Standard (control)**	0.8 ± 0.7 a	54.3 ± 3.2	26.2 ± 7.3 c	7,824.1 ± 2,179 c	**Standard (control)**	0.8 ± 0.3 c	57.5 ± 2.6 ab	46.4 ± 7.3 a	13,837.0 ± 2,206 a
**Leaf removal west**	0.6 ± 0.6 b	55.3 ± 4.0	29.9 ± 8.6 b	8,899.4 ± 2,585 b	**Leaf removal west**	0.7 ± 0.3 c	57.8 ± 2.4 a	42.3± 6.5 b	12,620.5 ± 1,934 b
**STD-UV-B**	0.4 ± 0.3 c	53.9 ± 2.8	31.4 ± 9.0 b	9,330.5± 2,695 b	**LR (-UV-B, -PAR)**	1.4 ± 0.7 a	56.7 ± 3.2 c	32.6 ± 4.6 c	9,740.8 ± 1,372 c
**LRW-UV-B**	0.5 ± 0.3 bc	54.7 ± 2.6	35.6 ± 8.4 a	10,614.2 ± 2,523 a	**LR (-UV-B, 2xUHI)**	0.9 ± 0.3 b	56.9 ± 2.5 cb	46.5 ± 9.1 a	13,895.6 ± 2,738 a
***Significance***	***	ns	***	***	***Significance***	***	***	***	***

Means in columns followed by a different letter are significantly different within one season. Mass conversion based on percent recovery of proanthocyanidin by phloroglucinolysis; mDP, mean degree of polymerization; %G, percentage galloylation; %P, percentage prodelphinidins; avMM, average molecular mass. STD (shaded/control); LRW (leaf removal west); STD with decreased UV-B radiation (STD-UV-B); LRW with decreased UV-B radiation (LRW-UV-B); LR (-UV-B, -PAR) (leaf removal with decreased UV-B radiation and 2xOp50 UV sheets added on both sides of the bunch zone); LR-UV-B, 2xUHI (leaf removal with decreased UV-B radiation and 2xUHI UV sheets added on both sides of the bunch zone). Different letters indicate significant differences at p ≤ 0.05, 0.01, and 0.001, respectively; ns, not significant.

Grape skin tannin also differs from grape seed tannin, as it has a lower percentage of galloylation (**Tables S7** and **S8**). Kazuya and Goto-Yamamoto (2008) reported that shading favored galloylation in grape skins. In our results, not all the shaded treatments had consistently higher galloylation percentages compared with the more exposed treatments. Additionally, the percentage prodelphinidins varied between 53.9% and 55.3% in 2010/2011 and 56.7% and 57.5% in 2011/2012 (**Table 9**). These prodelphinidin percentages are consistent with what have been reported by others (De Freitas et al., 2000; Gregan et al., 2012).

### Correlation Between Grape Seed and Skin Composition and Temperature and Light

The relationship between grape seed and grape skin compositions was examined by a hierarchical cluster analysis during ripening (**Figures 2A, B**). Shared clusters indicate the strength of the relationship between the grape seeds and skin concentration and content. In most cases, clusters were formed between the respective seed and skin fraction concentrations and contents. However, this was not consistent over the two seasons, indicating a greater seasonal effect on berry development. A similar scenario was observable in the compositional data by phloroglucinolysis (**Figures 2C, D**). Clusters were formed between the different terminal and extension subunits, but the clustering was not consistent over the two seasons.

### Flavonol Evolution During Ripening

**Figure 3:**
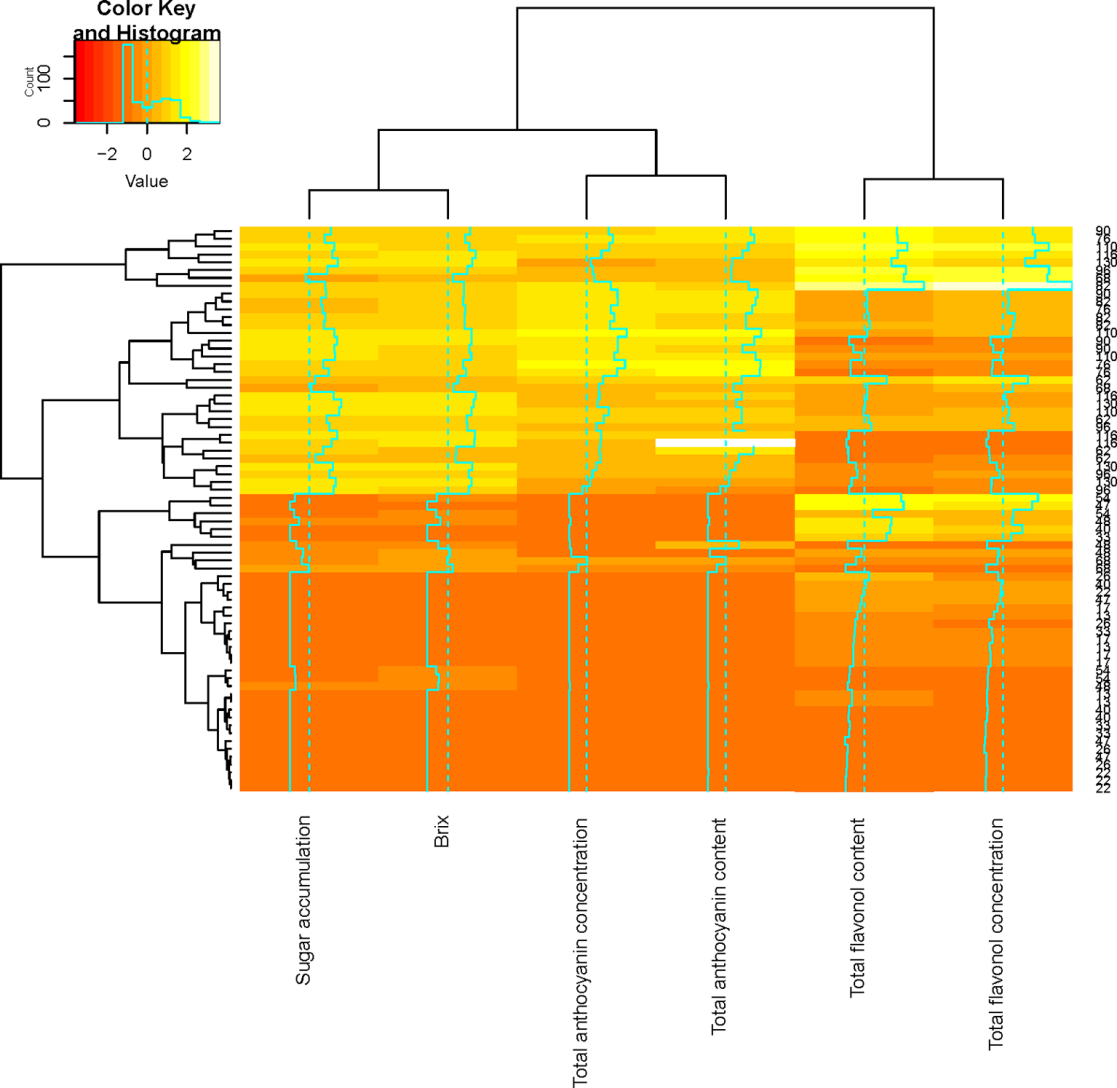
Correlation analysis showing clustered image maps of the correlation between flavonol and anthocyanin composition during ripening.

Flavonol accumulation commenced after fruit set until harvest in both seasons (**Figures 3**). Throughout both seasons, quercetin-3-*O*-glucoside and quercetin-3-*O*-glucuronide were the most abundant flavonol glycosides, while quercetin-3-*O*-rutinoside and quercetin-3-*O*-galactoside were present in smaller quantities (data not shown). This contradicts the findings of Mattivi et al. (2006) who reported that myricetin is the major flavonol in Cabernet Sauvignon. In both seasons, the patterns of accumulation were characterized by an increase after fruit set reaching a maximum 4 and 5 weeks post-véraison in 2010/2011 and 2011/2012, respectively, followed by small fluctuations in 2010/2011 or a decrease in 2011/2012 (**Figure 3**). Downey et al. (2006) also found a decrease in flavonols per berry 2–4 weeks after véraison in both exposed and shaded fruit within one season, while the flavonol content fluctuated from véraison until harvest in the other seasons.

Flavonol concentration and content were higher in the LRW treatment when compared with the other treatments (**Figure 3**). Similar patterns of accumulation were seen in the STD and LRW treatments in 2011/2012. The treatments with the UV-B exclusion sheets had the lowest flavonol concentration and content throughout ripening for both seasons (**Figure 3**). Our results also indicate a clear seasonal impact on flavonol evolution during ripening and are due to the significant impact of the season on the light quality and quantity. This is in agreement with the findings of several other authors (Price et al., 1995; Haselgrove et al., 2000; Spayd et al., 2002; Downey et al., 2004) who reported that shaded fruit had lower flavonol glucosides at harvest or during berry development in, respectively, Cabernet Sauvignon, Shiraz, and Merlot noir. Martínez-Lüscher et al. (2019) suggested that flavonol profile is a reliable indicator to assess canopy architecture and exposure of red wines to solar radiation. Flavonol concentration and content clustered together over the two seasons (**Figure 3**). This indicates that flavonol synthesis is independent within the grape berry skin.

Our data suggest that UV-B radiation plays an important role in the photo-protection of the berry against light exposure. Increased sunlight radiation resulted in high levels of UV exposure, leading to increased flavonol levels. Consequently, the flavonol concentration and content are dependent on the light quality. This in agreement with other studies describing that fruit exposed to different light qualities had higher flavonol glucosides (Crippen and Morrison, 1986; Downey et al., 2003). The latter phenomenon was also confirmed by Flint et al. (1985) and Berli et al. (2011). These authors suggested that flavonols act as UV screening compounds, protecting the plant tissue from the light damage during berry ripening. In this way, the accumulation of phenols takes place in the epidermal cell vacuoles of leaf tissue and grape berries, thereby protecting the photosynthetic mesophyll tissue (Flint et al., 1985; Macheix et al., 2005).

**Figure 4:**
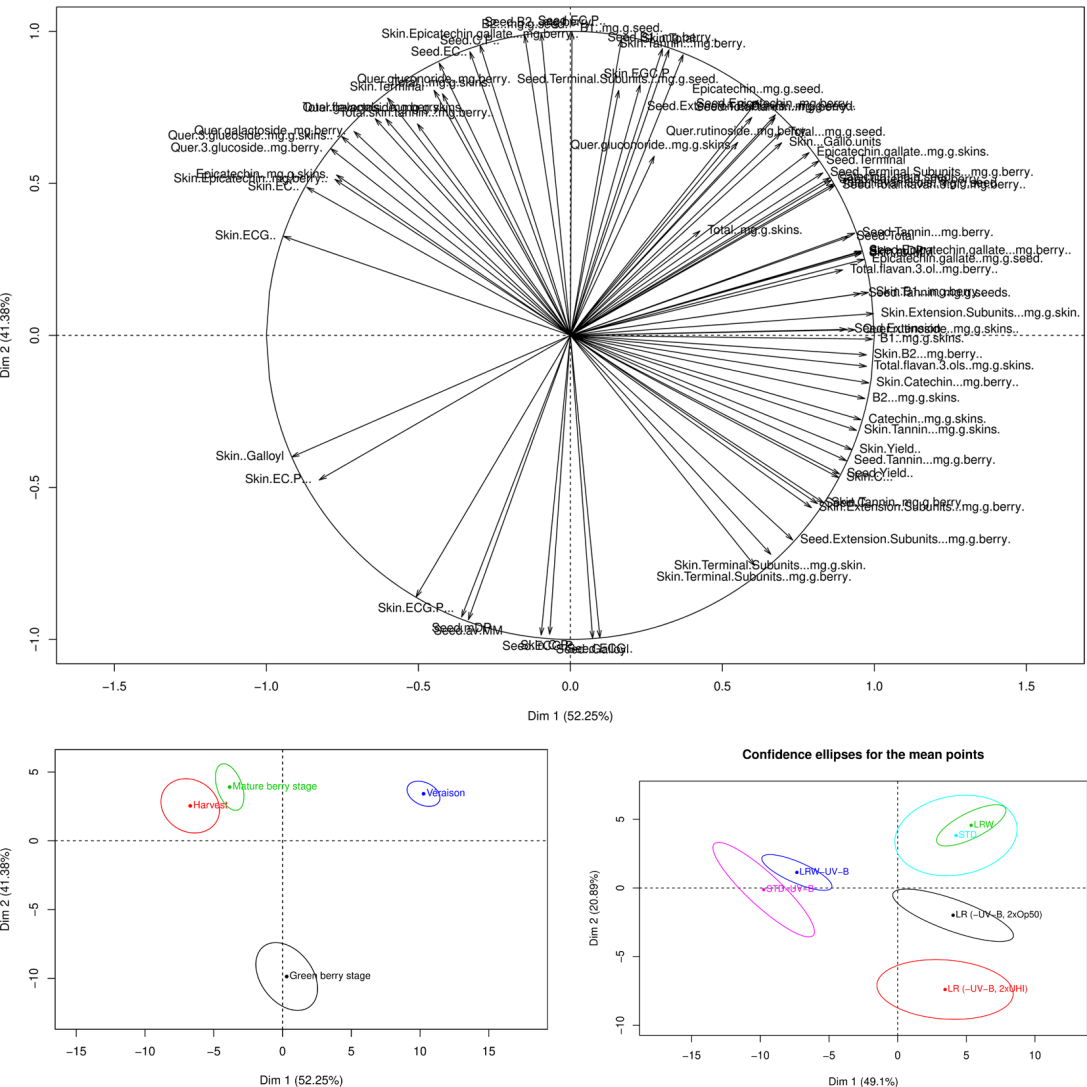
PCA loading and scores plots of the tannins and flavonols throughout both seasons. **(A)** PCA loading plot of tannins and flavonols. **(B)** PCA scores plot according to the variable distribution by phenological stage. **(C)** PCA scores plot according to the variable distribution by treatment.

Differences were observed in the analysis of the individual flavonoid compounds. Although ANOVAs were performed to assess the statistical impact of the different variables on the overall and individual flavonoid development, PCA plots were also generated to determine the cumulative effect from all the different variables on the overall phenological stage and treatment. The PCA loading plot shows the variable distribution according to the tannin and flavonols (**Figure 4A**). The green berry, mature, and harvest stages were mainly distributed along PC1 (52.5%) and the véraison stage along PC2 (41.38%) (**Figure 4B**). The majority of the changes in the grape seed, skin tannins, and flavonols occurred at véraison in both seasons. STD, LRW, LR (-UV-B, 2xOp50), and LR (-UV-B, 2xUHI) were mainly on the right side of PC1 axis (49.1%) (**Figure 4C**). Additionally, tannin and flavonol compounds from STD-UV-B and LRW-UV-B were distributed along PC2 (20.89%) (**Figure 4C**). From these results, it is clear that the tannin and flavonol compounds in both seasons were consistent in the STD and LRW treatments with the -UV-B treatments from both seasons responded differently. The -UV-B treatments in 2011–2012 have shown to have a bigger impact on the tannins and flavonols.

### Evolution of Anthocyanin Composition During Ripening

Mono-glucosides, acetyl-glucoside, and coumaroyl-glucoside derivatives of delphinidin, petunidin, peonidin, and malvidin were determined in both seasons (data not shown). The accumulation of the individual anthocyanins commenced at véraison, at 48 DAA and 68 DAA, in 2010/2011 and 2011/2012, respectively (**Figure 3**), which is in agreement with the findings of other researchers (Ryan and Revilla, 2003; Downey et al., 2006; Mori et al., 2007). In the total anthocyanin pool, mono-glucoside was the predominant form, while acetyl-glucoside and coumaroyl-glucoside forms were present in lower proportions (data not shown). Malvidin-3-*O*-glucoside was the dominant anthocyanin, and malvidin-3-*O*-acetyl glucoside was the major acylated anthocyanin in all treatments in both seasons (data not shown).

The trend of anthocyanin accumulation differed between the two seasons, as shown in **Figure 3**. The 2010/2011 season was characterized by an increase in anthocyanin concentration and content from véraison and a decrease from 90–116 DAA (**Figure 3A**). The 2011/2012 season was characterized by an increase after véraison between 68 and 82 DAA, a decrease between 83 and 96 DAA, followed by another increase from 96 to 110 DAA, and a decrease from 110 to 130 DAA. The STD and LRW treatments (treatments that were consistent over the two seasons) showed similar concentrations and contents at 48 DAA with a maximum at 76 DAA and similar levels at 116 DAA in the 2010/2011 season (**Figure 3**). Overall, there was no significant difference in the anthocyanin concentration and content in both the STD and LRW treatments for both seasons. The treatments with the UV-B exclusion sheets did not vary significantly from the STD and LRW treatments in 2010/2011. The mean anthocyanin concentration and content of the STD-UV-B and LRW-UV-B treatments in 2010/2011 were also similar. However, in the 2011/2012 season, the shaded LR (-UV-B, 2xOp50) had the highest overall concentration and content when than do the other treatments. The LR (-UV-B, 2xUHI) treatment had the lowest concentration and content as shown in **Table 10**, while it had the highest light exposure in addition to UV-B exclusion. Conflicting treatment results indicate that the season had a significant impact. Overall, there was no significant difference in the anthocyanin concentration and content in both the STD and LRW treatments for both seasons (**Table 10**). The mean anthocyanin concentration and content of the STD-UV-B and LRW-UV-B treatments in 2010/2011 were also similar (**Table 10**).

**Table 10:** The mean anthocyanin concentration and content in 2010/2011 and 2011/2012.

Treatment	Anthocyanin concentration (mg/g skin)	Anthocyanin content (mg/berry)
**2010/2011**		
**Standard (control)**	1.17	0.15
**Leaf removal west**	1.12	0.14
**STD-UV-B**	1.18	0.15
**LRW-UV-B**	1.25	0.14
***Significance***	ns	ns
**2011/2012**		
**Standard (control)**	1.22	0.13 ab
**Leaf removal west**	1.11	0.12 ab
**LR (-UV-B, 2xOp50)**	1.30	0.16 a
**LR (-UV-B, 2xUHI)**	0.92	0.10 b
***Significance***	ns	*

Each value represent the mean of five replicates at eight sampling dates in 2010/2011 and 10 sampling dates in 2011/2012. Means in columns followed by a different letter are significantly different within one season. STD (shaded/control); LRW (leaf removal west); LR-UV-B, 2xOp50 (leaf removal with decreased UV-B radiation and 2xOp50 UV sheets added on both sides of the bunch zone); LR-UV-B, 2xUHI (leaf removal with decreased UV-B radiation and 2xUHI UV sheets added on both sides of the bunch zone). Different letters indicate significant differences at p ≤ 0.05, 0.01, and 0.001, respectively; ns, not significant.

The individual anthocyanin composition in 2010/2011 was not significantly different for most of the anthocyanins among treatments. However, in 2011/2012, significant differences were observed in all the derivatives except for petunidin coumaroyl-glucoside. This indicates that the higher temperatures experienced in 2010/2011 had a larger impact rather than PAR. This also resulted in similar concentrations and contents of anthocyanins in the STD and LRW treatments in 2010/2011 and 2011/2012, respectively. The second season (2011/2012) was cooler, resulting in shifts in the anthocyanin profiles, confirming the findings of other authors who found vintage effects to play an important role in anthocyanin composition (Crippen and Morrison, 1986; Gao and Cahoon, 1994).

Ryan and Revilla (2003) suggested that anthocyanin fingerprint of a grape cultivar grown in a given location changed slightly from year to year, probably because of anthocyanin biosynthesis modulation by weather conditions during ripening. Our results did not show a clear trend in anthocyanin accumulation in grape related to a different light exposure. Inconsistent treatment effects indicated that seasonal (climatic) impact was greater than any impact due to treatment.

Numerous studies investigated the impact of temperatures on anthocyanins (Crippen and Morrison, 1986; Gao and Cahoon, 1994; Spayd et al., 2002; Mori et al., 2005; Mori et al., 2007). Other studies have also found significant differences between seasons (Brossaud et al., 1999; Spayd et al., 2002), whereas Mazza et al. (1999) reported a minimal influence of the season in Cabernet franc, Merlot, and Pinot noir due to an atypical growing season over three seasons. Recently, Šebela et al. (2017) suggested that the phenolic concentration in Chardonnay is a function of the sunlight intensities preceding the sampling date during the ripening season.

**Figure 5:**
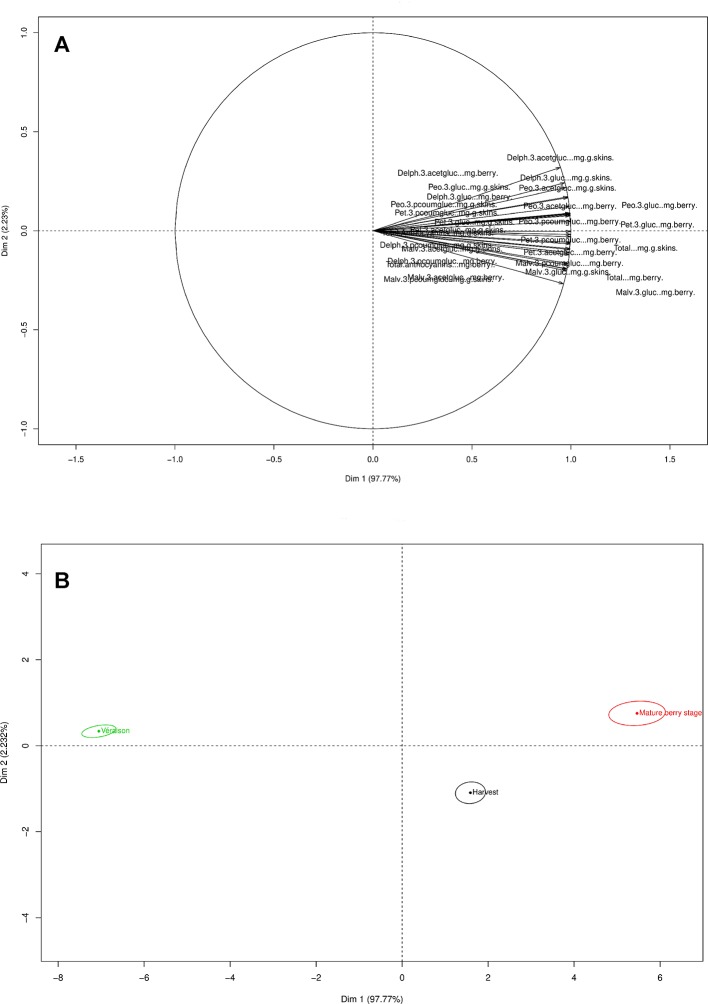
PCA loading and scores plots of the anthocyanins throughout both seasons. **(A)** PCA loading plot of anthocyanins. **(B)** PCA scores plot according to the variable distribution by phenological stage.

The PC1 from the PCA loadings for anthocyanin’s explained 97.77% of the variance, while PC2 described (2.32%) of the variance (**Figure 5A**). Anthocyanin development was impacted by the phenological stage and developed during the mature berry stage.

### Correlation Between Flavonol and Anthocyanin Composition and Temperature and Light

The relationship between flavonol and anthocyanin composition as well as sugar accumulation was examined by a hierarchical cluster analysis during ripening (**Figure 3**). Shared clusters indicate the strength of the relationship between the flavonol, anthocyanin composition, and sugar accumulation. Anthocyanin composition and sugar accumulation formed a cluster, while flavonol concentration and content formed another cluster (**Figure 3**). Our results indicate that anthocyanin accumulation and that of sugar take place concurrently. These findings correspond with those of Conde et al. (2007) who suggested that sugar and anthocyanin accumulation commence at stage 3 of berry development. A second cluster was formed by the flavonol concentration and content during ripening (**Figure 3**). This suggests that flavonol synthesis occurs independently in the grape berry.

## Conclusions

This study highlighted the complexity of working under vineyard conditions to investigate the complex interaction of abiotic factors on berry metabolites. The novelty of our study involves the work being conducted under actual vineyard conditions in South Africa, which experiences high levels of UV radiation, while other studies were based on experimental setups (Spayd et al., 2002; Berli et al., 2008). This assisted to answer another research question, which was to assess the potential effect of the treatments on Cabernet Sauvignon wine style (data not shown). The results suggested that tannin evolution is dependent on the prevailing light quality/quantity during berry development in a particular season, while leaves and laterals at the bunch zone seemed not to impact flavan-3-ol metabolism under the seasonal conditions studied. The bulk of both seed and skin monomers, dimers, and tannin was synthesized just after fruit set and reached a maximum at véraison, after which it decreased in both seasons. The post-véraison decrease of the seed and skins monomers, dimers, and tannin concentration and content is ascribed to a reduction in the extractability of the tannin post-véraison. The skin tannin increases/decreases observed during berry growth could be ascribed to the pattern of expression of flavonoid pathway genes reported by Boss et al. (1996).

We hypothesize that the light quality and quantity are a potential factor affecting the final skin total tannin concentration and content. Brown et al. (2005) found that low UV-B rates result in UV-B stimulation of some genes that are involved in a wide range of processes that are responsible for flavonoid and phenolic production (UV protection). Skin tannins, therefore, play a photo-protective role within the berry. This study highlights the importance of including seed number data and dry mass data to enhance interpretation. The applied treatments in this study did not introduce significant temperature differences. Treatments did result in differences in light quantity and quality, which had only a marginal impact on skin flavan-3-ol synthesis and no effect on seed tannin. In the case of skin tannin, there was a hint of increased skin tannin with light exposure, but this was only visible in the 2010/2011 seasons, indicating that seasonal variability had a larger impact than the individual treatments applied to alter the light quantity and quality. Therefore, from a vineyard perspective, seasonal differences have a large impact on berry seed and skin tannin composition and extractability; additional studies over several seasons are needed.

Flavonol and anthocyanin evolution is dependent on the prevailing light quality/quantity and temperatures during berry development in a particular season. Flavonol accumulation was significantly impacted in treatments that restricted UV-B light in the bunch zone, resulting in significant decreases in flavonol biosynthesis in both seasons studied. Therefore, it can be concluded that the light quality is the main abiotic driver of skin flavonol biosynthesis regulation. Anthocyanin concentration and content were largely influenced by the season and not the treatments applied, suggesting a synergistic influence of both light quantity and temperature with limited impact due to UV-B exclusion.

From a research perspective, working under controlled conditions should help to better understand the effect of abiotic factors. At the same time, it should be taken into account that working in vineyard conditions has consequences for the wine quality and style.

## Data Availability

All datasets[GENERATED/ANALYZED] for this study are included in the article and the **Supplementary Material**.

## Author Contributions

AD, AO, and JR-d-S conceptualized and planned the study. EB and AO implemented the viticultural treatments. EB maintained the viticultural treatments and was responsible for berry sampling. EB was responsible for the extraction of grape seed and skin tannin and RP-HPLC analysis and phloroglucinolysis. EB, AO, JR-d-S, and AD drafted the original manuscript, and all authors contributed and finalized the publication.

## Funding

We gratefully acknowledge the Wine Industry Network for Expertise and Technology (Winetech) grant number WW EW 10-02 and Technology and Human Resources for Industry Programme (THRIP) grant number (TP2010072000042) for financial support.

## Conflict of Interest Statement

The authors declare that the research was conducted in the absence of any commercial or financial relationships that could be construed as a potential conflict of interest.

## Abbreviations

avMM, Average molecular mass; B1, Dimer Ec-(4β-8)-Cat; B2, Dimer Ec-(4β-8)-Ec; DAA, Days after anthesis; DD, Degree days; HPLC-DAD, High-performance liquid chromatography diode array; LOD, Limit of detection; LOQ, Limit of quantification; LRW, Leaf removal west side of the bunch zone just after flowering; LRW-UV-B, Leaf removal west and UV sheet (Perspex^®^ Opal 050) on the western side of the bunch; LR (-UV-B, 2xOp50), Leaf removal on both sides of the canopy (in the bunch zone) and (Perspex^®^ Opal 050) on both sides of the bunch zone; LR (-UV-B, 2xUHI), Leaf removal on both side of the canopy (in the bunch zone) and UV sheet (UHI) extruded clear acrylic was used on both sides of the bunch zone; mDP, Mean degree of polymerization; PAR, Photosynthetically active radiation; RP-HPLC, Reversed-phase high-performance liquid chromatography; STD, Standard (control) treatment; STD-UV-B, Control treatment and UV sheet (Perspex^®^ Opal 050) on the western side of the bunch; TSS, Total soluble solids; PAL, Phenylalanine ammonia-lyase; UV, Ultraviolet radiation.
