# Supplementary material for: Grape Flavonoid Evolution and Composition Under Altered Light and Temperature Conditions in Cabernet Sauvignon (Vitis vinifera L.)
Source: Front Plant Sci. 2019 Nov 8;10:1062. doi: 10.3389/fpls.2019.01062 (PMC6874162; doi:10.3389/fpls.2019.01062)
Supplement: Supplementary file 1 [file DataSheet_1.pdf]

Table S1. The experimental layout created by leaf removal and UVB attenuation and optical properties of ‘Perspex’ ® and acrylic UV sheets.

**2010/2011**			
**Shaded (Control) (STD)**	**Exposed - Leaf Removal West (LRW)**	**STD with decreased UV-B radiation (STD-UV-B)**	**LRW with decreased UV-B radiation (LRW-UV-B)**
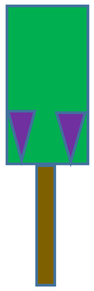	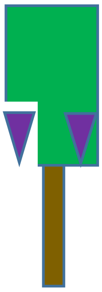	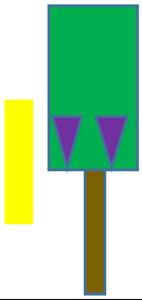	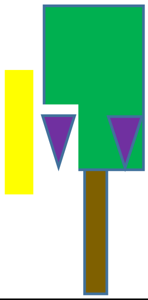
**2011/2012**			
**Shaded (Control) (STD)**	**Exposed - Leaf Removal West (LRW)**	**LR (-UV-B,-PAR) (Shaded without leaves and laterals) Leaf removal with decrease in PAR and UV-B radiation and 2xOp50 UV-sheets added on both sides of the bunch zone**	**LR(-UV-B, 2xUHI) Leaf removal with decreased UV-B radiation and 2xUHI UV-sheets added on both sides of the bunch zone**
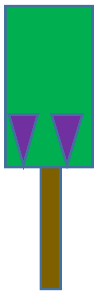	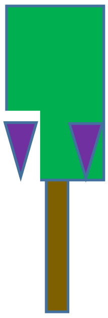	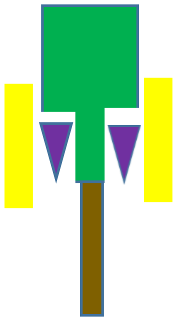	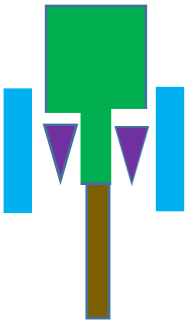

**2010/2011**									
Perspex ® Opal 050	**Visible (380-780 nm)**		**Solar (350-2100 nm)**					Shading coefficient	UV Elimi-nation
	Light transmission %	Light reflection %	Total elimination %	Direct reflection %	Absorption %	Direct transmission %	Total Trans-missio n %		
	27	42	59	31	38	31	41	0.47	99
**2011/2012**									
Extruded high impact acrylic	89	78	12	8	6	86	88	1.0	99

Table S2. Sampling dates (for phenolic analysis) and days after anthesis for 2010/2011 and 2011/2012 seasons (Blancquaert, 2015).

Sampling and days after anthesis dates			
**2010/2011 season**		**2011/2012 season**	
**Sampling dates**	**Days after anthesis**	**Sampling dates**	**Days after anthesis**
7 December 2010	13	14 December 2011	26
11 December 2010	17	21 December 2011	33
16 December 2010	22	28 December 2011	40
11 January 2011	48	04 January 2012	47
25 January 2011	62	11 January 2012	54
08 February 2011	76	25 January 2012	68
22 February 2011	90	08 February 2012	82
20 March 2011	116	22 February 2012	96
		06 March 2012	110
		26 March 2012	130

Table S3. Temperature classes at different phenological stages.

	Minimum temperature				Mean temperature				Maximum temperature			
**2010-2011**	**Green berry stage**	**Véraison**	**Mature berry stage**	**Harvest**	**Green berry stage**	**Véraison**	**Mature berry stage**	**Harvest**	**Green berry stage**	**Véraison**	**Mature berry stage**	**Harvest**
**STD**	16,37	15,02	16,35	16,85	23,42	23,55	23,83	23,85	31,84	32,83 b	33,34 b	34,31 b
**LRW**	16,21	14,93	16,13	16,11	23,74	24,08	24,53	23,94	34,04	35,00 ab	36,95 a	36,93 a
**STD-UV-B**	16,10	14,63	16,03	16,39	23,85	24,30	24,27	24,18	33,17	35,23 a	34,29 b	33,48 ab
**LRW-UV-B**	16,21	14,93	16,34	16,85	23,74	24,08	23,94	23,85	34,04	35,00 ab	33,94 b	34,31 b
p-value	ns	ns	ns	ns	ns	ns	ns	ns	ns	0,024396	0,000001	0,000001
	**Minimum temperature**				**Mean temperature**				**Maximum temperature**			
**2011-2012**	**Green berry stage**	**Véraison**	**Mature berry stage**	**Harvest**	**Green berry stage**	**Véraison**	**Mature berry stage**	**Harvest**	**Green berry stage**	**Véraison**	**Mature berry stage**	**Harvest**
**STD**	13,56	18,85	13,76	12,33	23,86	25,47	23,59	23,72	41,96 a	39,99 a	39,17 a	44,24 a
**LRW**	13,51	18,76	13,80	12,51	23,08	25,45	23,18	22,89	35,78 b	37,13 b	38,16 a	40,97 b
**LR(-UV-B,2xOp50)**	13,26	18,62	13,56	12,31	24,22	26,42	23,97	23,62	39,93 a	40,89 a	39,43 a	39,91 b
**LR(-UV-B,2xUHI)**	13,23	18,55	13,55	12,28	22,92	25,10	22,56	22,21	35,42 b	35,4878 c	33,84 b	35,62 c
p-value	ns	ns	ns	ns	ns	ns	ns	ns	0,000000	0,000000	0,000000	0,000000

Each value represents the mean of 5 replicates. STD (Shaded/Control); LRW (Leaf Removal West); STD-UV-B (STD with decreased UV-B radiation); LRW-UV-B (LRW with decreased UV-B radiation). LR (-UV-B,-PAR) (Leaf removal with decreased UV-B radiation and 2xOp50 UV-sheets added on both sides of the bunch zone); LR-UV-B, 2xUHI (Leaf removal with decreased UV-B radiation and 2xUHI UV-sheets added on both sides of the bunch zone). Different letters indicate significant differences at ($p \leq 0.05$, 0.01, and 0.001, respectively; ns: not significant).

Table S4. Berry parameters at harvest of 2010/2011 and 2011/2012 (Blancquaert, 2015).

Treatment	Total soluble solids	Fresh mass (g)	Sugar per berry
**2010/2011**			
STD	20.5 b	60.3 b	290.9 b
LRW	22.4 a	58.3 b	282.9 b
STD-UV-B	22.4 a	52.1 c	285.2 b
LRW-UV-B	22.9 a	63.1 a	316.7 a
Significance	**	***	***
**2011/2012**			
STD	23.9 a	72.7 a	348.0 a
LRW	23.1 bc	68.4 b	327.3 b
LR (-UV-B, 2xOp50)	23.1 b	68.4 b	289.7 d
LR(-UV-B, 2xUHI)	22.6 c	63.4 c	305.1 c
Significance	***	***	***

Each value represents the mean of 5 replicates (±) standard deviation. Treatments: STD (Shaded/Control); LRW (Leaf Removal West); STD-UV-B (STD with decreased UV-B radiation); LRW-UV-B (LRW with decreased UV-B radiation); LR-UV-B, 2xOp50 (Leaf removal with decreased UV-B radiation and 2xOp50 UV-sheets added on both sides of the bunch zone); LR-UV-B, 2xUHI (Leaf removal with decreased UV-B radiation and 2xUHI UV-sheets added on both sides of the bunch zone). Different letters indicate significant differences at ($p \leq 0.05$, 0.01, and 0.001, respectively; ns: not significant).

Table S5. Compositional and structural characterization of seed extracts during ripening in 2010/2011 done by phloroglucinolysis (Blancquaert, 2015).

DAA	Treatment	Terminal units [a]			Extension units [a]			mDP	%G[b]	avMM[b]	Proanthocyanidins
		C	EC	ECG	C	EC	ECG				
13	**Standard (Control)**	79.63 ± 3.91	14.89 ± 3.09	5.46 ± 0.97	12.01 ± 1.59	86.95 ± 1.83	1.03 ± 0.30	5.9 ± 0.7	1.8 ± 0.5 a	1726 ± 205 a	11.2 ± 5.5 a
	**Leaf Removal West**	83.71 ± 1.73	12.74 ± 1.38	3.53 ± 0.64	12.17 ± 0.43	87.1 ± 0.36	0.731 ± 1.25	5.5 ± 0.5	1.2 ± 0.25 b	1611 ± 162 ab	12.5 ± 2.6 a
	**STD-UV-B**	88.98 ± 1.60	12.01 ± 1.00	nd	12.13 ± 0.90	88.79 ± 1.46	nd	5.0 ± 0.4	0 c	1466 ± 106 b	11.06 ± 5.5 a
	**LRW-UV-B**	79.63 ± 3.91	14.89 ± 3.09	5.46 ± 0.97	12.01 ± 1.59	88.79 ± 1.46	nd	5.9 ± 0.7	1.8 ± 0.5 a	1726 ± 205 a	11.2 ± 2.4 a
Significance		***	***ns***	***	***ns***	***ns***	***ns***	ns	ns	***ns***	***ns***
17	**Standard (Control)**	81.52 ± 1.57	14.06 ± 1.23	4.40 ± 0.87	10.78 ± 0.58	88.2 ± 0.64	1.01 ± 0.49	8.1 ± 0.3 a	1.4 ± 0.5 a	2360 ± 99.3 a	16.2 ± 2.2 a
	**Leaf Removal West**	81.65 ± 1.37	14.18 ± 1.49	4.16 ± 0.50	11.23 ± 0.61	87.70 ± 0.40	1.06 ± 0.31	7.1 ± 0.6 b	1.4 ± 0.3 a	2081.2 ± 194 b	14.6 ± 3.1 a
	**STD-UV-B**	86.03 ± 0.83	13.31 ± 1.51	0.65 ± 0.96	11.11 ± 0.54	88.73 ± 0.54	0.1 ± 0.15	6.5 ± 0.9 b	0.2 ± 0.2 b	1896 ± 276 c	13.2 ± 2.3 a
	**LRW-UV-B**	81.52 ± 1.57	14.06 ± 1.23	4.4 ± 0.87	10.78 ±0.58	88.73 ± 0.54	0.1 ± 0.15	8.1 ± 0.3 a	1.4 ± 0.5 a	2360 ± 99.3 a	16.2 ± 2.2 a
Significance		***ns***	***ns***	***ns***	***ns***	***ns***	***ns***	**	**	***	***ns***
22	**Standard (Control)**	72.71 ± 2.73	16.19 ± 1.39	11.09 ± 3.01	10.05 ± 0.14	86.08 ± 1.30	3.86 ± 1.37	9.2 ± 0.2 a	4.6 ± 1.5 a	2720 ± 92.7 a	19.8 ± 0.8 a
	**Leaf Removal West**	75.31 ± 2.37	16.68 ± 0.75	8 ± 3.01	10.26 ± 0.43	87.25 ± 0.80	2.48 ± 1.05	9.1 ± 0.4 a	3.1 ± 1.1 a	2692 ± 145.1 a	17.6 ± 1.45 b
	**STD-UV-B**	82.82 ± 2.19	18.05 ± 0.67	nd	11.36 ± 0.47	90.43 ± 4.02	0.1 ± 0.14	7.6 ± 0.2 b	0.13 ± 0.12 b	2192 ± 92.3 b	11.48 ± 2.5 c
	**LRW-UV-B**	72.71 ± 2.73	16.19 ± 1.39	11.09 ± 0.87	10.05 ± 0.14	90.43 ± 4.02	0.1 ± 0.14	9.2 ± 0.2 a	4.6 ± 1.5 a	2720 ± 92.7 a	19.8 ± 0.8 a
Significance		***	***ns***	***	***ns***	**	*	***	***	***	***
48	**Standard (Control)**	64.73 ± 3.24	31.08 ± 2.21	4.18 ± 5.07	12.08 ± 1.39	85.41 ± 2.06	2.5 ± 3.21	2.0 ± 0.7 b	1.7 ± 0.9 a	588.1 ± 205 b	29.6 ± 3.4 b
	**Leaf Removal West**	66.25 ± 1.97	28.54 ± 1.65	5.2 ± 0.76	10.37 ± 0.47	86.53 ± 0.59	3.09 ± 0.44	3.1 ± 0.3 a	3.7 ± 0.5 b	915.1 ± 97.7 a	34.7 ± 2.5 a
	**STD-UV-B**	67.08 ± 1.79	30.96 ± 1.74	1.94 ± 0.69	11.15 ± 0.97	87.94 ± 0.85	0.9 ± 0.301	2.7 ± 0.2 a	1.3 ± 0.4 b	791.4 ± 62.4 a	27.46 ± 1.9 b
	**LRW-UV-B**	67.01 ± 1.53	29.95 ± 1.11	3.02 ± 1.18	11.21 ± 0.62	87.94 ± 0.85	0.9 ± 0.301	3.0 ± 0.3 a	2.02 ± 0.8 b	876 ± 107.5 a	28.1 ± 1.35 b
Significance		***ns***	***ns***	***ns***	**	***ns***	***ns***	***	**	*	**
62	**Standard (Control)**	58.57 ± 1.55	37.12 ± 1.49	4.3 ± 2.34	10.83 ± 0.53	85.56 ± 2.19	3.6 ± 2.22	3.8 ± 0.5 a	3.8 ± 2.2 a	1123.9 ±157.9 a	33.5 ± 4.6 a

	**Leaf Removal West**	62 ± 3.00	34.5 ± 2.41	3.49 ± 0.94	11.56 ± 0.79	85.95 ± 0.74	2.48 ± 0.44	3.7 ± 0.5 a	2.7 ± 0.5 a	1108 ± 150 a	26.2 ± 3.6 b
	**STD-UV-B**	60 ± 3.35	39.22 ± 3.51	0.76 ± 0.46	12.03 ± 0.37	87.58 ± 0.21	0.38 ± 0.26	2.9 ± 0.2 b	0.5 ± 0.3 b	863. 1 ± 72.4 b	18.1 ± 2.6 c
	**LRW-UV-B**	60.92 ± 0.70	37.74 ± 1.25	1.33 ± 1.42	12.36 ± 0.51	87.58 ± 0.21	0.38 ± 0.26	3.0 ± 0.3 b	1.1 ± 1.1 b	886 .7 ± 101.8 b	20.4 ± 2.5 c
Significance		***ns***	*	***ns***	***ns***	***ns***	***ns***	**	*	*	***

Each value represents the mean of 5 replicates (±) standard deviation in units of mg/g seed tannin extract. STD (Shaded/Control); LRW (Leaf Removal West); STD-UV-B (STD with decreased UV-B radiation); LRW-UV-B (LRW with decreased UV-B radiation). [a]Percent composition of proanthocyanidin subunits (in moles) C, (+)-catechin; EC, (−)-epicatechin; ECG, (−)-epicatechin-3-O-gallate. mDP, mean degree of polymerization; %G, percentage galloylation; avMM, average molecular mass ; nd, not detected. Different letters indicate significant differences at ($p \leq 0.05$, 0.01, and 0.001, respectively; ns: not significant).

Table S6. Compositional and structural characterization of seed extracts during ripening in 2011/2012 done by phloroglucinolysis (Blancquaert, 2015).

DAA	Treatment	Terminal units [a]			Extension units [a]			mDP	%G[b]	avMM[b]	Proanthocyanidins
		C	EC	ECG	C	EC	ECG				
26	Standard (Control)	54.8 ± 2.9	11.9 ± 1.0	33.3 ± 2.0	8.6 ± 0.7	77 ± 1.5	14.4 ± 0.9	7.8 ± 0.8 a	16.8 ± 0.9 ab	2450 ± 257.4 a	31.3 ± 3.7 a
	Leaf Removal West	56.3 ± 0.5	11.4 ± 0.4	32.3 ± 0.7	8.1 ± 0.4	77.6 ± 0.3	14.3 ± 0.4	7.4 ± 0.2 a	16.7 ± 0.3 b	2347.2 ± 66.0 a	28.6 ± 1.4 a
	LR (-UV-B,-PAR)	54.2 ± 1.4	10.6 ± 1.1	31.2 ± 7.2	8.3 ± 0.6	71.9 ± 8.8	14 ± 3.9	7.5 ± 0.6 a	17.9 ± 1.1 a	2353.5 ± 173.2 a	28.8 ± 2.5 a
	LR (-UV-B, 2xUHI)	53.7 ± 4.4	11.6 ± 1.0	29.4 ± 6.6	8.3 ± 0.9	73.1 ± 9.5	12.7 ± 2.9	7.9 ± 0.5 a	16.5 ± 0.7 b	2485 ± 150.9 a	29.9 ± 3.3 a
Significance		***ns***	***ns***	***ns***	***ns***	***ns***	***ns***	***ns***	***ns***	***ns***	***ns***
33	Standard (Control)	49.6 ± 2.5	13.3 ± 1.4	37.2 ± 2.1	8.9 ±0.6	77.6 ± 1.2	13.5 ± 0.9	7.6 ± 0.9 a	16.6 ± 0.9 ab	2384 ± 7.2 a	21.2 ± 7.2 a
	Leaf Removal West	52.3 ± 1.0	13.0 ± 1.6	34.8 ± 1.1	9.2 ± 0.5	77.7 ± 0.4	13.1 ± 0.6	7.5 ± 0.2 a	15.9 ± 0.7 b	2342.6 ± 51.0 a	23.3 ± 3.6 a
	LR (-UV-B,-PAR)	51.8 ± 1.2	12.1 ± 0.4	36.1 ± 0.8	9.8 ± 0.4	75.8 ± 0.7	14.4 ± 0.4	6.8 ± 0.3 a	17.6 ± 0.4 a	2145.4 ± 83.3 a	23.0 ± 2.8 a
	LR (-UV-B, 2xUHI)	51.7 ± 2.5	12.7 ± 1.8	32.0 ± 8.8	9.7 ± 0.9	77. 4 ± 1.5	12.8 ± 0.6	7.5 ± 0.6 a	15.9 ± 0.8 b	2330.2 ± 178.7 a	25.3 ± 3.3 a
Significance		***ns***	***ns***	*	***ns***	***ns***	***ns***	***ns***	*	***ns***	***ns***
40	Standard (Control)	46.9 ± 1.1	16.0 ± 0.8	37.2 ± 0.6	9.4 ± 0.5	78.8 ± 0.5	11.9 ± 0.4	7.1 ± 0.3 b	15.4 ± 0.3 a	2218.9 ± 84.5 b	24.7 ± 3.3 b
	Leaf Removal West	50.7 ± 1.2	13.8 ± 0.6	35.5 ± 1.1	9.6 ± 0.3	79.2 ± 0.6	11.2 ± 0.7	7.5 ± 0.2 a	14.4 ±0.5 c	2330.9 ± 68.8 a	26.2 ± 0.7 b
	LR (-UV-B,-PAR)	46.6 ± 0.7	15.8 ± 1.0	37.6 ± 0.4	9.8 ± 0.2	79.1 ± 0.5	11.1 ± 0.4	7.0 ± 0.2 b	14.9 ± 0.3 b	2181.5 ± 80.9 bc	27.4 ± 0.8 ab
	LR (-UV-B, 2xUHI)	49.4 ± 1.1	15.4 ± 0.7	35.2 ± 1.0	9.9 ± 0.3	79.4 ± 0.4	10.7 ± 0.2	6.8 ± 0.2 b	14.3 ± 0.3 c	2130.5 ± 54.8 c	29.7 ± 1.7 a
Significance		*	***ns***	*	***ns***	***ns***	***ns***	***	***	***	.
47	Standard (Control)	48.8 ± 2.9	19.8 ± 0.7	31.4 ± 2.5	9.6 ± 0.2	78.6 ± 0.5	11.8 ± 0.4	4.5 ± 0.6 b	16.2 ± 0.2 ab	1406.2 ± 202.5 b	40.7 ± 6.1 a
	Leaf Removal West	48.8 ± 1.5	18.7 ± 1.3	32.5 ± 2.3	9.3 ± 0.3	79 ± 0.3	11.7 ± 0.4	5.2 ± 0.6 a	15.7 ± 0.3 bc	1637 ± 190.9 a	35.7 ± 4.3 bc
	LR (-UV-B,-PAR)	46.4 ± 1.1	20.2 ± 1.7	33.4 ± 2.2	9.9 ± 0.3	78.1 ± 0.45	12 ± 0.3	4.8 ± 0.5 ab	16.4 ± 0.3 a	1506.9 ± 156.8 ab	32.0 ± 2.6 b
	LR (-UV-B, 2xUHI)	51.4 ± 4.2	17.1 ± 2.4	28.9 ± 0.8	9.4 ± 0.6	79.2 ± 0.3	11 ± 0.4	4.6 ± 0.3 b	15.3 ± 0.4 c	1436 ± 107.2 b	39.1 ± 2.1 ab
Significance		*	***ns***	***ns***	***ns***	***	***ns***	***ns***	***	ns	*
54	Standard (Control)	62.5 ± 2.0	33.6 ± 2.4	4.2 ± 3.5	11.4 ± 1.5	85.9 ± 1.4	1.6 ± 1.6	3.2 ± 0.3 b	2.3 ± 2.2 b	949.1 ± 81.4 b	25.3 ± 4.5
	Leaf Removal West	66 ±3.7	31.5 ± 3.4	2.4 ± 1.4	11.3 ± 0.6	87.9 ± 0.6	0.8 ± 0.6	3.2 ± 0.3 b	1.3 ± 0.8 b	945.6 ± 78.6 b	19.5 ± 4.9
	LR (-UV-B,-PAR)	64.4 ± 1.6	33.6 ± 1.5	2.0 ± 1.0	12.5 ± 0.8	87 ± 0.6	0.5 ± 0.3	3.4 ± 0.35 ab	0.9 ± 0.5 b	994.3 ± 102.8 b	22.1 ± 9.5

	LR (-UV-B, 2xUHI)	60.1 ± 9.9	25.2 ± 5.0	10.3 ± 9.8	10.4 ± 1.7	79.7 ± 8.5	4.3 ± 4.6	3.6 ± 0.2 a	7.5 ± 7.2 a	1096. 3 ± 99.1 a	35.0 ± 12.4
Significance		*	ns	*	*	ns	ns	*	*	*	**
68	Standard (Control)	58.7 ± 3.0	37.8 ± 3.4	3.5 ± 1.4	11.9 ± 1.2	86.5 ± 0.8	1.6 ± 0.9	3 ± 0.2 a	2.2 ± 1.0 a	885.3 ± 67.4 a	23. 0 ± 2.8 b
	Leaf Removal West	59.2 ± 3.7	37.8 ± 3.5	3.0 ± 1.9	12.1 ±1.0	69 .2 ± 38.2	1.4 ± 1.1	3.1 ± 0.3 a	1.9 ± 1.3 a	876.9 ± 82.4 a	23.1 ± 5.3 b
	LR (-UV-B,-PAR)	63.4 ± 4.3	30 ± 1.6	2.7 ± 1.2	12.1 ± 1.2	77.8 ± 8	1.3 ± 0.6	2.5 ± 0.2 b	2.7 ± 1.4 a	736.3 ±75.3 b	26.6 ± 4.4 b
	LR (-UV-B, 2xUHI)	63.1 ± 1.6	33.4 ± 1.8	3.6 ± 2.2	13.1 ± 0.6	85.4 ± 0.9	1.5 ±1.1	3.1 ± 0.3 a	2.1 ± 1.4 a	878.4 ± 92.7 a	25.5 ± 4.2
Significance		*	ns	ns	ns	ns	ns	*	ns		
82	Standard (Control)	57.5 ± 1.7	39.3 ± 1.7	3.1 ± 1.2	12.2 ± 0.6	86.3 ± 0.4	1.5 ± 0.6	3.6 ± 0.4 b	1.9 ± 0.7 a	1066 ± 111.9 b	22.0 ± 4.0 a
	Leaf Removal West	58.8 ± 1.5	38.8 ± 2.0	2.4 ± 1.1	11.2 ± 1.2	86.9 ± 2.2	1.9 ± 1.6	4 ± 0.5 b	2.0 ± 1.6 a	1170.3 ± 145.2 b	24.0 ± 4.5 a
	LR (-UV-B,-PAR)	58.5 ± 2.3	38.6 ± 2.9	2.9 ± 1.0	12.3 ± 0.6	86.2 ± 0.7	1.5 ± 0.6	3.6 ± 0.1 b	1.9 ± 0.7 a	1060.5 ± 43.7 b	21.8 ± 2.1 a
	LR (-UV-B, 2xUHI)	58.3 ± 1.9	37.6 ± 3.3	3.5 ± 1.9	11.6 ± 0.7	84 ± 5.7	1.7 ± 1.2	4.5 ± 0.3 a	2.5 ± 1.6 a	1320.8 ± 87.3 a	21.7 ± 1.6 a
Significance		ns	ns	ns	ns	ns	ns	***	ns	**	ns
96	Standard (Control)	56.0 ± 3.5	41.6 ± 3.1	2.3 ± 1.0	12.5 ± 1.1	86.1 ± 0.5	1.3 ± 0.7	4.1 ± 0.4 b	1.5 ± 0.7 ab	1194.3 ± 125 b	18.9 ± 2.8 ab
	Leaf Removal West	56.8 ± 1.5	39.5 ± 2.2	3.7 ± 1.7	10.7 ± 0.9	87 ± 1.7	2.4 ± 1.5	4.9 ± 0.2 a	2.6 ±1.5 a	1433.3 ± 62.9 a	21.6 ± 3.0 a
	LR (-UV-B,-PAR)	58.4 ± 1.5	40.3 ± 2.9	2.0 ± 1.0	12.9 ± 0.7	87.9 ± 7.3	1.0 ± 1.7	4.1 ± 0.2 b	1.2 ± 0.8 b	1163.4 ± 69.0 b	17.6 ± 1.9 bc
	LR (-UV-B, 2xUHI)	58.0 ± 2.1	39.4 ± 2.6	2.6 ± 0.9	12.2 ± 0.6	86.5 ± 0.9	1.3 ± 0.6	4.7 ± 0.3 a	1.5 ± 0.6 ab	1373.3 ± 81.2 a	15.0 ± 3.2 c
Significance		ns	ns	ns	***	ns	ns	***	ns	***	**
110	Standard (Control)	54.7 ± 2.1	41.4 ± 3.5	3.9 ± 2.1	11.8 ± 0.6	85.7 ± 1.4	2.4 ± 1.9	4.6 ± 0.4 a	2.8 ± 1.9 a	1340 ± 143.2 a	25.1 ± 4.2 a
	Leaf Removal West	55.9 ± 0.7	40.1 ± 2.0	4.0 ± 1.9	10.9 ± 0.7	86.9 ± 1.5	2.1 ± 1.2	4.8 ± 0.1 a	2.5 ± 1.3 a	1416.7 ± 32.5 a	22.6 ± 1.3 ab
	LR (-UV-B,-PAR)	57.9 ± 3.6	41.0 ± 3.9	1.7 ± 1.2	13.6 ± 0.7	88.2 ± 4.6	0.8 ± 0.8	3.9 ± 0.1 b	0.6 ± 0.3 b	1142.3 ±81.8 b	18.6 ± 3.0 b
	LR (-UV-B, 2xUHI)	56.3 ± 1.4	41.5 ± 1.7	2.2 ± 0.7	12.7 ± 0.5	86.4 ± 0.6	0.8 ± 0.5	4.6 ± 0.2 a	1.1 ± 0.5 ab	1335.4 ± 80.2 a	20.0 ± 2.2 b
Significance		ns	ns	ns	***	ns	ns	**	ns	**	*
130	Standard (Control)	54.9 ± 2.2	41.8 ± 1.8	2.8 ± 1.0	11.9 ± 0.3	84.4 ± 3.6	1.8 ± 0.9	4.4 ± 0.2 b	2.1 ± 0.8 a	1300 ± 76 b	20.3 ± 3.1 a
	**Leaf** Removal West	56.4 ± 1.7	40.6 ± 1.5	3.0 ± 1.6	12.3 ± 1.0	85.7 ± 1.2	1.9 ± 1.3	4.5 ± 0.6 ab	2.1 ± 1.4 a	1313.9 ± 171.9 ab	16.0 ± 4.5 ab
	LR (-UV-B,-PAR)	60.8 ± 3.7	37.4 ± 32.6	3.1 ± 0.7	12.2 ± 1.2	85.8 ± 9.0	4.1 ± 4.2	4.4 ± 0.6 b	2.1 ± 0.6 a	1280.4 ± 170.7 b	18.3 ± 2.2 ab
	LR (-UV-B, 2xUHI)	54.6 ± 1.7	42.0 ± 2.4	3.4 ± 1.5	11.6 ± 0.5	86.3 ± 0.9	2.1 ± 1.2	5.1 ± 0.4 a	2.3 ± 1.2 a	1494.1 ± 135 a	15.4 ± 2.3 b
Significance		*	ns	ns	ns	ns	ns	ns	ns	ns	*

Each value represents the mean of 5 replicates (±) standard deviation in units of mg/g seed tannin extract.). STD (Shaded/Control); LRW (Leaf Removal West); LR (-UV-B,-PAR) (Leaf removal with decreased UV-B radiation and 2xOp50 UV-sheets added on both sides of the bunch zone); LR-UV-B, 2xUHI (Leaf removal with decreased UV-B radiation and 2xUHI UV-sheets added on both sides of the bunch zone). [a]Percent composition of proanthocyanidin subunits (in moles) C, (+)-catechin; EC, (−)-epicatechin; ECG, (−)-epicatechin-3-O-gallate. mDP, mean degree of polymerization; %G, percentage galloylation; avMM, average molecular mass ; nd, not detected. Different letters indicate significant differences at ($p \leq 0.05$, 0.01, and 0.001, respectively; ns: not significant).

Table S7. Compositional and structural characterization of skin extracts during ripening in 2010/2011 done by phloroglucinolysis (Blancquaert, 2015).

DAA	Treatment	Terminal units [a]			Extension units [a]				mDP	%G	% P	avMM	Proanthocyanidins
		C	EC	ECG	C	EC	ECG	EGC					
13	**Standard (Control)**	94.0 ± 5.5	5.95 ± 5.55	nd	2.52 ± 0.19	45.52 ± 1.19	0.15 ± 0.09	51.802 ± 1.39	22.5 ± 2.3	0.15 ± 0.1 a	49.5 b	6735.8 ± 701 a	12.1 ± 6.1 a
	**Leaf Removal West**	95.8 ± 5.66	4.13± 5.66	nd	2.65 ± 0.47	41.39 ± 2.4	0.08± 0.07	55.86 ± 2.76	22.4 ± 5.2	0.08 ± 0.1 b	53.3 a	6661.4 ± 1541 a	11 ± 3.4 a
	**STD-UV-B**	94.96 ± 4.6	5.03 ± 4.66	nd	2.52 ± 0.19	45.52 ± 1.19	0.15 ± 0.09	51.8 ± 1.39	22.8 ± 2.4	0.10 ± 0.1ab	49.5 b	6754 ± 712 a	7.3 ± 4.1 a
	**LRW-UV-B**	94.0 ± 5.5	5.95 ± 5.55	nd	2.52 ± 0.19	45.52 ± 1.19	0.15 ± 0.09	51.8 ± 1.39	22.5 ± 2.3	0.15 ± 0.1 ab	49.5 b	6735.8 ± 701 a	12.1 ± 6.1 a
Significance		***ns***	***ns***	***ns***	***ns***	***ns***	***ns***	*ns*	*ns*	**ns**	*ns*	*ns*	*ns*
17	**Standard (Control)**	91.4 ± 0.46	8.53 ± 0.46	nd	1.93± 0.16	41.4 ± 1.49	0.20 ± 0.07	56.45 ± 1.61	35.3 ± 1.85 a	0.16 ± 0.1 ab	54.9 a	10497 ± 555.7 a	20.5 ± 2.5 a
	**Leaf Removal West**	100	nd	nd	2.81 ± 0.39	37.5 ± 2.86	nd	59.67 ± 2.96	21.2 ± 3.5 b	0 ± 0 c	56.8 a	6302 ± 1049 b	4.7 ± 1.9 c
	**STD-UV-B**	97.8 ± 4.8	2.16 ± 4.83	nd	2.1 ±0.29	39.4± 2.57	0.05 ± 0.08	58.30 ± 2.84	31.6 ± 8.13 a	0.05 ± 0.1bc	56.4 a	9416.7 ± 2428.3 a	12.6 ± 3.2 b
	**LRW-UV-B**	91.4 ± 0.46	8.53 ± 0.46	nd	1.93± 0.16	41.4 ± 1.49	0.20 ± 0.07	56.45 ± 1.61	35.3 ± 1.85 a	0.16 ± 0.1 ab	54.8 a	10497 ± 555.7 a	20.5 ± 2.5 a
Significance		***ns***	***ns***	***ns***	*	***ns***	***ns***	***ns***	***	***	***ns***	**	***
22	**Standard (Control)**	100	nd	nd	2.58 ± 0.52	41.6 ± 1.30	0.41 ± 0.11	55.39 ± 1.18	20.5 ± 4.3	0.4 ± 0.1 a	52.6 b	6089.7 ± 1301.4 a	6.5 ± 3.2 a
	**Leaf Removal West**	100	nd	nd	2.43 ± 0.25	39.0 ± 1.78	0.03 ± 0.07	58.49 ± 1.97	24 ± 2.9	0.03 ± 0.1 c	56 a	7140.2 ± 869 a	7.7 ± 2.0 a
	**STD-UV-B**	86.9 ± 2.12	13 ± 2.12	nd	2.55 ± 0.36	42.2 ± 1.5	0.24 ± 0.07	54.99 ± 1.84	22.7 ± 3.1	0.23 ± 0.1 b	52.5 b	6742.2 ± 934 a	8.4 ± 3.5 a
	**LRW-UV-B**	100	nd	nd	2.58 ± 0.52	41.6 ± 1.30	0.41 ± 0.11	55.39 ± 1.18	20.5 ± 4.3	0.4 ± 0.1 a	52.6 b	6089.7 ± 1301.4 a	6.5 ± 3.2 a
Significance		***ns***	***ns***	***ns***	***ns***	***ns***	***ns***	***ns***	***ns***	**	***	***ns***	***ns***
48	**Standard (Control)**	95.9 ± 1.0	nd	nd	1.50 ± 0.18	40.9 ± 0.85	0.44 ± 0.08	57.12 ± 0.92	38.8 ± 2.0 b	0.53 ± 0.1 a	55.6 b	11546.5 ± 610 b	18.4 ± 2.3 a
	**Leaf Removal West**	89.4 ± 1.7	10.5 ± 1.7	nd	1.76 ± 0.29	35.8 ± 3.47	0.45 ± 0.84	61.97 ± 2.46	38.2 ± 4.1 b	0.44 ± 0.1 a	60.3 a	11397.8 ± 1272.6 b	16.6 ± 5.3 ab
	**STD-UV-B**	100	nd	nd	1.72 ± 0.18	39.6 ± 3.04	0.14 ± 0.08	58.46 ± 3.17	42.1 ± 5.1 ab	0.14 ± 0.1 a	57.1 b	12523.6 ± 1540.7 ab	13.4 ± 3.2 b
	**LRW-UV-B**	78.1 ± 32.7	2.3 ± 3.2	nd	1.32 ± 0.56	32.7 ± 16.56	0.24 ± 0.18	46.74 ± 22.63	44.7 ± 2.8 a	0.31 ± 0.1 a	56.3 b	13300.8 ± 846 a	17.1 ± 2.1 ab
Significance		**	***ns***	***ns***	***ns***	***ns***	***ns***	*	*	***ns***	*	*	***ns***
62	**Standard (Control)**	57.7 ± 11.4	23.4 ± 7.95	nd	1.70 ± 0.23	40.7 ± 4.76	0.47 ± 0.70	57.34 ± 5.01	23.7 ± 6.5 c	1.5 ± 1.4 a	54.8 a	7086.2 ± 1909 c	7.1 ± 1.9 b

	**Leaf Removal West**	70.2 ± 4.8	22.8 ± 3.16	nd	1.71 ± 0.13	41.36 ± 1.26	1.7 ± 0.08	55.75 ± 1.36	43.9 ± 4.5 a	1.3 ± 0.1 ab	54.5 a	13127.6 ± 1368.0 a	13.9 ± 2.2 a
	**STD-UV-B**	68.6 ± 7.2	21.6 ± 7.49	nd	1.78 ± 0.24	41.8 ± 0.36	0.30 ± 0.06	56.11 ± 0.32	33.5 ± 8.3 b	0.60 ± 0.1 ab	54.3 a	9975.2 ± 2482.3 b	9.5 ± 2.8 b
	**LRW-UV-B**	89.7 ± 2.12	8.07 ± 18.06	nd	1.77 ± 0.14	40.4 ± 1.35	0.19 ± 0.07	57.48 ± 1.22	42.1 ± 1.6 a	0.44 ± 0.1 b	56.2 a	12543.5 ± 481.6 a	8.3 ± 2.0 b
Significance		*	*	***ns***	***ns***	***ns***	***	***ns***	***	*	***ns***	***	**
**76**	**Standard (Control)**	65.1 ± 7.39	13.03 ± 1.55	nd	1.72 ± 0.09	41.4 ± 2.27	0.25 ± 0.14	56.61 ± 2.36	23.3 ± 4.4 d	1.2 ± 0.5 a	54.1 b	6974.8 ± 1302.3 c	6.3 ± 1.3 c
	**Leaf Removal West**	54.9 ± 5.2	31.1 ± 8.01	nd	1.66 ± 0.20	41.3 ± 2	1.09 ± 0.20	55.92 ± 2.37	31.9 ± 1.9 b	1.5 ± 0.3 a	54.1 b	9539.6 ± 586.4 b	11.7 ± 1.6 a
	**STD-UV-B**	66.79 ± 5.21	23.42 ± 5.38	nd	1.93 ± 0.12	42.2 ± 1.8	0.27 ± 0.16	55.52 ± 1.84	27.6 ± 27.6 c	0.62 ± 0.1 b	53.5 b	8213.6 ± 83.1 c	9.3 ± 1.5 b
	**LRW-UV-B**	80.9 ± 6.83	3.22 ± 7.2	nd	1.72 ± 0.13	39.6 ± 2.2	0.11 ± 0.03	58.39 ± 2.19	40.5 ± 2.9 a	0.6 ± 0.3 b	56.9 a	12066.7 ± 880.6 a	10.8 ± 1.7 ab
Significance		***	***	***ns***	***ns***	***ns***	***	***ns***	***	**	*	***	**
**90**	**Standard (Control)**	62.15 ± 9.6	18.60 ± 10.5	nd	1.81 ± 0.19	37.8 ± 1.43	0.18 ± 0.09	60.21 ± 1.52	25 ± 3.5 b	0.94 ± 0.11 ab	57.8 a	7483.6 ± 1057.2 b	7.5 ± 0.8 b
	**Leaf Removal West**	59.3 ± 3.91	29.1 ± 6.87	nd	1.87 ± 0.34	43.5 ± 7.8	0.29 ± 0.17	54.27 ± 8.36	24 ± 3.3 b	0.77 ± 0.2 b	51.9 a	7151.2 ± 979.6 b	10.1 ± 2.3 a
	**STD-UV-B**	78.7 ± 7.18	nd	nd	1.88 ± 0.25	42.1 ± 1.05	0.38 ± 0.07	55.56 ± 1.19	35 ± 3.2 a	0.81 ± 0.04 ab	55.8 a	10.436.7 ± 970.6 a	8.7 ± 0.8 ab
	**LRW-UV-B**	77.6 ± 1.7	29.12 ± 6.8	nd	1.76 ± 0.17	40.5 ± 2.31	0.36 ± 0.11	56.4 ± 1.74	38.6 ± 1.1 a	0.97 ± 0.11 a	54 a	11525.2 ± 343.6 a	8.7 ± 0.6 ab
Significance		*	***	***ns***	***ns***	***ns***	***ns***	***ns***	***	*	***ns***	***	ns
**116**	**Standard (Control)**	67.0 ± 6.8	9.89 ± 5.67	nd	1.34 ± 0.12	40.11 ± 2.08	0.57 ± 0.27	57.95 ± 2.10	20.6 ± 2.1 c	1.66 ± 0.2 a	55.1 a	6178.9 ± 620.5 c	4.9 ± 0.4 c
	**Leaf Removal West**	67.3 ± 10	18.74 ± 10.72	nd	1.59 ± 0.12	40.7± 2.62	0.36 ± 0.06	57.32 ± 2.74	33.1 ± 4.7 b	0.8 ± 0.1 b	55.6 a	9875.1 ± 1386.1 b	11 ± 1.6 a
	**STD-UV-B**	83.7 ± 0.75	nd	nd	1.87 ± 0.17	44.6 ± 0.88	0.34 ± 0.11	53.16 ± 0.97	37 ± 3.9 ab	0.7 ± 0.1 b	51.7 b	11015 ± 1158.7	7.8 ± 1.4 b
	**LRW-UV-B**	79.5 ± 4.4	3.15 ± 7.05	nd	1.57 ± 0.36	42.6 ± 2.86	0.26 ± 0.07	57.6 ± 4.01	40.8 ± 4.5 a	0.7 ± 0.1 b	55.1 a	12154 ± 1330.5 a	8.0 ± 1.1 b
Significance		***ns***	*	***ns***	***ns***	***ns***	***ns***	***ns***	***	*	***	***	***

Each value represents the mean of 5 replicates (±) standard deviation in units of mg/g skin tannin extract. STD (Shaded/Control); LRW (Leaf Removal West); STD-UV-B (STD with decreased UV-B radiation); LRW-UV-B (LRW with decreased UV-B radiation). [a]Percent composition of proanthocyanidin subunits (in moles) C, (+)-catechin; EC, (−)-epicatechin; ECG, (−)-epicatechin-3-O-gallate. mDP, mean degree of polymerization; %G, percentage galloylation; avMM, average molecular mass ; nd, not detected. Different letters indicate significant differences at (p ≤ 0.05, 0.01, and 0.001, respectively; ns: not significant).

Table S8. Compositional and structural characterization of skin extracts during ripening in 2011/2012 vintage done by phloroglucinolysis (Blancquaert, 2015).

DAA	Treatment	Terminal units [a]			Extension units [a]				mDP	%G	%P	avMM	Proanthocyanidins
		C	EC	ECG	C	EC	ECG	EGC					
26	Standard (Control)	89.4 ± 7.8	8.4 ± 5.8	1.3 ± 0.3	1.3 ± 0	42.7 ± 1.4	1.3 ± 0.1	54.7 ± 1.4	46.1 ± 11.9 a	53.4 ± 1.6 a	1.3 ± 0.1 bc	13751.6 ± 3575.3 a	22.6 ± 4.4 a
	Leaf Removal West	94.4 ± 1.5	4.2 ± 2.1	1.4 ± 0.7	1.4 ± 0.1	41.8 ± 2.0	1.1 ± 0.1	55.3 ± 1.6	47. 3 ± 6.6 a	54.4 ± 1.9 a	1.2 ± 0.2 c	14105.8 ± 1995.3 a	22.8 ± 2.2 a
	LR (-UV-B,-PAR)	62.6 ± 2.3	4.8 ± 0.7	32.6 ± 2.6	1.4 ± 0.1	44.6 ± 1.4	1.1 ± 0.1	52.9 ± 1.5	28 ± 2.2 b	51.0 ± 1.5 b	2.2 ± 0.1 a	8387.6 ± 653.4 b	23.4 ± 2.1 a
	LR (-UV-B, 2xUHI)	92.6 ± 1.2	5.6 ± 1.4	1.7 ± 0.3	1.5 ± 0.1	43.5 ± 0.9	1.4 ± 0.1	53.6 ± 1.0	51.7 ± 6.8 a	52.5 ± 1.1 ab	1.4 ± 0.1 b	15429.4 ± 2024.2 a	19.8 ± 2.7 a
Significance		***	*ns*	***	*ns*	*ns*	*ns*	*ns*	**	***	*	**	ns
33	Standard (Control)	90.8 ± 1.3	7.3 ± 1.1	1.8 ± 0.3	1.3 ± 0.1	40.6 ± 1.8	1.2 ± 0.1	56.9 ± 2.0	55.7 ± 4.5 a	55.9 ± 2.0 a	1.2 ± 0.1 b	16656.2 ± 1347.5 a	17.1 ± 3.8 a
	Leaf Removal West	91.2 ± 1.2	6.9 ± 1.7	1.9 ± 0.5	1.4 ± 0.2	40.3 ± 1.4	1.2 ± 0.1	57.1 ± 1.5	51.7 ± 6.9 a	56.0 ± 1.3 a	1.2 ± 0.1 b	15447.5 ± 2043.8 a	16.3 ± 4.7a
	LR (-UV-B,-PAR)	53.7 ± 2.3	3.8 ± 0.5	42.5 ± 2.7	1.6 ± 0.1	41.6 ± 1.6	1.2 ± 0	55.7 ± 1.6	32 ± 1.7 b	53.9 ± 1.6 a	2.5 ± 0.1 a	9619.9 ± 507.2 b	14.9 ± 3.0 a
	LR (-UV-B, 2xUHI)	90.4 ± 1.8	7.1 ± 0.4	2.5 ± 1.0	1.4 ± 0.2	41.0 ± 1.7	1.4 ± 0.1	56.2 ± 1.7	56.4 ± 7.7 a	55.2 ± 1.8 a	1.4 ± 0.1 b	16848.1 ± 2315.1 a	14.9 ± 3.4 a
Significance		***	*ns*	***	*ns*	*ns*	*ns*	*ns*	***	*ns*	***	***	
40	Standard (Control)	91.3 ± 2.18	8.5 ± 1.6	1.6 ± 0.6	1.2 ± 0.1	39.5 ± 1.7	1.0 ± 0	58.3 ± 1.7	47.3 ± 0.7 b	57.1 ± 1.7 a	1.0 ± 0 b	14124 ± 199.7 b	24.5 ± 2.9 a
	Leaf Removal West	90.6 ± 1.8	7.6 ± 1.7	1.7 ± 0.8	1.2 ± 0.1	39.3 ± 1.6	1.1 ± 0.1	58.4 ± 1.6	46.8 ± 2.5 b	57.2 ± 1.6 a	1.1 ± 0.1 b	13978.2 ± 749.5 b	23.4 ± 2.2 a
	LR (-UV-B,-PAR)	53.0 ± 1.7	4.1 ± 1.4	42.9 ± 2.3	1.3 ± 0	40.5 ± 1.3	1.1 ± 0.1	57.1 ± 1.3	34.4 ± 1.6 c	55.4 ± 1.2 a	2.3 ± 0.2 a	10341.4 ± 476.5 c	16.2 ± 1.8 b
	LR (-UV-B, 2xUHI)	91.9 ± 1.6	6.5 ± 1.4	1.6 ± 0.5	1.2 ± 0.1	40.0 ± 1.1	1.2 ± 0.1	57.6 ± 1.2	54.0 ± 3.0 a	56.5 ± 1.3 a	1.2 ± 0 b	16127.3 ± 895.0 a	17.9 ± 1.9 b
Significance		***	*ns*	***	*ns*	*ns*	*ns*	*ns*	***	*ns*	***	***	***
47	Standard (Control)	96.2 ± 1.0	3.2 ± 1.1	0.6 ± 0.1	1.4 ± 0.1	38.5 ± 1.0	0.5 ± 0.1	59.6 ± 1.0	51.5 ± 5.1 a	58.5 ± 1.0 ab	0.5 ± 0 c	15362.9 ± 1532.6 a	14.0 ± 2.5 ab
	Leaf Removal West	95.7 ± 1.2	3.7 ± 1.3	0.7 ± 0.2	1.3 ± 0.1	37.3 ± 2.3	0.4 ± 0.2	61.0 ± 2.4	44.1 ± 3.6 b	59.5 ± 2.2 a	0.4 ± 0.2 c	13164.0 ± 1078.8 b	15.7 ± 2.5 a
	LR (-UV-B,-PAR)	70.1 ± 11.2	2.3 ± 0.7	2.3 ± 0.3	1.4 ± 0.2	38.5 ± 1.6	0.5 ± 0.4	59.3 ± 1.8	36.6 ± 4.9 b	57.9 ± 1.9 ab	1.6 ± 0.4 a	10972.0 ± 1466.0 c	11.3 ± 1.9 b
	LR (-UV-B, 2xUHI)	93.7 ± 1.1	4.2 ± 0.8	2.1 ± 0.9	1.3 ± 0.1	40.0 ± 1.7	1.2 ± 0.1	57.5 ± 1.8	55.1 ± 5.8 a	56.5 ± 1.8 b	1.2 ± 0.1 b	16460.3 ± 1750.1 a	14.2 ± 2.8 ab

Significance		***	ns	ns	ns	ns	***	ns	**	ns	***	***	ns
54	Standard (Control)	97.0 ± 0.8	2.5 ± 0.9	0.5 ± 0.1	1.2 ± 0.1	36.4 ± 1.3	0.3 ± 0.1	62.0 ± 1.4	45.5 ± 2.2 a	60.7 ± 1.4 a	0.3 ± 0.1 b	13576.9 ± 649.5 a	18.1 ± 4.5 ab
	Leaf Removal West	88.5 ± 4.0	10.8 ± 4.4	0.7 ± 0.4	1.3 ± 0.1	36.9 ± 1.0	0.4 ± 0.1	61.6 ± 1.0	40.1 ± 3.9 b	60.1 ± 0.8 a	0.3 ± 0.1 b	11950.9 ± 1171.1 b	19.8 ± 2.5 a
	LR (-UV-B,-PAR)	87.2 ± 4.0	1.8 ± 0.8	14.5 ± 4.5	1.3 ± 0.1	36.8 ± 1.7	0.2 ± 0.1	61.6 ± 1.9	39.5 ± 1.5 b	60.1 ± 1.8 a	0.5 ± 0.1 a	11772.3 ± 444.8 b	18.4 ± 2.8 ab
	LR (-UV-B, 2xUHI)	96.7 ± 0.5	2.6 ± 0.4	0.7 ± 0.3	1.2 ± 0.1	38.3 ± 2.0	0.4 ± 0	60.2 ± 2.2	48.6 ± 5.2 a	59.0 ± 2.2 a	0.3 ± 0.1 b	14472.7 ± 1574.2 a	15.6 ± 1.5 b
Significance		ns	ns	***	ns	ns	ns	ns	**	ns	**	**	ns
68	Standard (Control)	67.6 ± 3.3	28.3 ± 2.0	4.1 ± 1.5	1.2 ± 0.1	37.7 ± 0.5	0.7 ± 0.1	60.5 ± 0.6	39.4 ± 1.7 a	58.9 ± 0.6 a	0.8 ± 0.1 a	11761.6 ± 486.4 a	13.0 ± 1.3 ab
	Leaf Removal West	72.7 ± 4.8	24.6 ± 4.6	2.8 ± 1.1	1.1 ± 0.1	37.8 ± 0.6	0.6 ± 0.2	60.5 ± 0.4	38.5 ± 4.2 ab	58.9 ± 0.3 a	0.6 ± 0.2 a	11481.3 ± 1250.0 ab	15.3 ± 0.6 a
	LR (-UV-B,-PAR)	63.9 ± 9.3	19.3 ± 4.2	18.8 ± 4.1	1.5 ± 0.1	37.8 ± 2.0	0.2 ± 0.1	60.5 ± 1.9	32.9 ± 3.6 b	58.7 ± 1.8 a	0.8 ± 0.3 a	9813.3 ± 1058.8 b	12.6 ± 3.0 b
	LR (-UV-B, 2xUHI)	75.2 ± 10.8	22.4 ± 9.9	2.4 ± 1.1	1.3 ± 0.1	37.7 ± 1.1	0.7 ± 0.1	60.3 ± 1.2	41.2 ± 5.7 a	58.8 ± 1.2 a	0.7 ± 0.2 a	12303.1 ± 1676.7 a	11.8 ± 1.1 b
Significance		ns	ns	***	***	ns	***	ns	*	ns	ns	*	ns
82	Standard (Control)	76.0 ± 1.0	20.6 ± 1.1	3.4 ± 0.8	1.2 ± 0.1	37.8 ± 1.0	0.4 ± 0.1	60.7 ± 1.0	42.7 ± 1.3 a	59.3 ± 1.0 a	0.4 ± 0.1 b	12729.7 ± 403.5 a	13.9 ± 2.6 a
	Leaf Removal West	76.8 ± 2.5	20.5 ± 2.5	2.7 ± 0.2	1.2 ± 0.1	37.5 ± 1.6	0.4 ± 0	60.9 ± 1.6	39.2 ± 2.5 b	59.3 ± 1.6 a	0.5 ± 0 b	11682.9 ± 738.6 b	14.1 ± 1.9 a
	LR (-UV-B,-PAR)	65.3 ± 2.6	19.8 ± 2.6	14.9 ± 2.8	1.1 ± 0	38.4 ± 2.0	0.3 ± 0.1	60.1 ± 2.0	33.6 ± 2.4 c	58.3 ± 1.9 a	0.8 ± 0.2 a	10033.1 ± 710.1 c	14.1 ± 2.2 a
	LR (-UV-B, 2xUHI)	74.7 ± 2.6	18.5 ± 2.2	6.8 ± 2.0	1.1 ± 0	37.2 ± 2.0	0.5 ± 0.1	61.1 ± 1.9	44.6 ± 1.4a	59.7 ± 1.8 a	0.7 ± 0.2 a	13302.7 ± 401.5 a	13.2 ± 0.9 a
Significance		ns	ns	***	ns	ns	ns	ns	***	ns	**	***	ns
96	Standard (Control)	86.0 ± 3.3	32.9 ± 18.4	7.9 ± 7.0	1.1 ± 0.1	40.1 ± 0.9	0.7 ± 0.1	58.1 ± 1.1	53.7 ± 5.0 a	57.0 ± 1.0 a	0.8 ± 0.1 a	16031.4 ± 1488.8 a	10.2 ± 1.7 a
	Leaf Removal West	61.2 ± 4.4	35.1 ± 5.2	3.7 ± 1.2	1.1 ± 0	39.1 ± 0.9	0.6 ± 0.1	59.2 ± 0.9	34.3 ± 4.3 c	57.5 ± 0.7 a	0.7 ± 0.1 a	10232.4 ± 1295.9 c	9.6 ± 1.4 a
	LR (-UV-B,-PAR)	51.3 ± 4.9	36.1 ± 4.2	12.6 ± 1.7	1.0 ± 0.1	38.1 ± 2.2	0.3 ± 0.1	60.7 ± 2.4	30.4 ± 5.2 c	58.6 ± 2.5 a	0.7 ± 0.2 a	9068.2 ± 1559.4 c	9.9 ± 1.6 a
	LR (-UV-B, 2xUHI)	65.3 ± 5.6	30.7 ± 6.4	4.0 ± 1.5	1.0 ± 0.1	38.9 ± 0.8	0.5 ± 0.1	59.5 ± 0.8	42.4 ± 6.0 b	58.1 ± 0.8 a	0.6 ± 0.1 a	12662.7 ± 1783.8 b	10.7 ± 1.8 a
Significance		*	***	***	ns	ns	***	ns	***	ns	Ns	***	ns
110	Standard (Control)	83.3 ± 3.3	11.4 ± 2.5	5.3 ± 1.4	1.2 ± 0.1	36.9 ± 1.1	0.6 ± 0	61.5 ± 1.1	46.8 ± 2.1 a	60.1 ± 1.0 a	0.6 ± 0.2 b	13982.9 ± 633.5 a	8.6 ± 1.2 c
	Leaf Removal West	73.4 ± 13.4	18.9 ± 9.7	7.7 ± 7.0	1.3 ± 0.1	36.8 ± 1.1	0.6 ± 0	61.4 ± 1.1	40.6 ± 7.5 ab	59.8 ± 1.3 ab	0.8 ± 0.3 b	12131.6 ± 2240.2 ab	10.9 ± 0.9 b
	LR (-UV-B,-PAR)	51.2 ± 7.6	32.0 ± 5.0	16.8 ± 6.1	1.2 ± 0	37.6 ± 1.5	0.6 ± 0.1	60.6 ± 1.3	29.7 ± 7.4 c	58.4 ± 1.0 bc	1.2 ± 0.4 a	8872.2 ± 2202.4 c	13.1 ± 1.7a
	LR (-UV-B, 2xUHI)	63.8 ± 7.0	29.2 ± 8.6	7.0 ± 1.7	1.2 ± 0.1	38.6 ± 0.7	0.6 ± 0	59.6 ± 0.7	37.8 ± 4.8 bc	58.0 ± 0.5 c	0.8 ± 0.1 b	11284.6 ± 1424.4 bc	13.3 ± 1.0 a

Significance		***	*	***	*ns*	*ns*	*ns*	*ns*	**	*	*	**	***
130	Standard (Control)	61.9 ± 1.9	31.7 ± 5.6	4.1 ± 0.9	1.2 ± 0	42.6 ± 1.6	0.7 ± 0	56 ± 1.5	34.9 ± 1.9 ab	54.3 ± 1.4 a	0.7 ± 0 b	10393.2 ± 559.2 ab	9.2 ± 0.6 ab
	Leaf Removal West	72.3 ± 13.0	20.3 ± 9.5	7.4 ± 7.8	1.3 ± 0.1	41.7 ± 2.5	0.6 ± 0.1	56.5 ± 2.6	40.4 ± 9.0 a	55.0 ± 2.6 a	0.7 ± 0.3 b	12030.6 ± 2684.6 a	8.8 ± 1.2 a
	LR (-UV-B,-PAR)	54.0 ± 8.0	32.2 ± 7.7	18.5 ± 4.7	1.3 ± 0.2	40.5 ± 3.4	0.5 ± 0.2	59.2 ± 12.5	28.6 ± 1.2 b	54.4 ± 2.5 a	1.2 ± 0.3 a	8528 ± 351.3 b	10.8 ± 1.6 bc
	LR (-UV-B, 2xUHI)	54.0± 3.8	43.2 ± 4.0	2.8 ± 0.6	1.2 ± 0	41.8 ± 1.8	0.6 ± 0.1	57.1 ± 2.7	33.8 ± 2.3 ab	54.8 ± 1.8 a	0.7 ± 0.1 b	10065.3 ± 683.7 ab	8.5 ± 0.8 c
Significance		***	***	***	ns	ns	ns	ns	*	ns	*	*	***

Each value represents the mean of 5 replicates (±) standard deviation in units of mg/g skin tannin extract.). STD (Shaded/Control); LRW (Leaf Removal West); LR (-UV-B,-PAR) (Leaf removal with decreased UV-B radiation and 2xOp50 UV-sheets added on both sides of the bunch zone); LR-UV-B, 2xUHI (Leaf removal with decreased UV-B radiation and 2xUHI UV-sheets added on both sides of the bunch zone). [a]Percent composition of proanthocyanidin subunits (in moles) C, (+)-catechin; EC, (−)-epicatechin; ECG, (−)-epicatechin-3-O-gallate; EGC, (−)-epigallocatechin. mDP, mean degree of polymerization; %G, percentage galloylation; avMM, average molecular mass ; nd, not detected. Different letters indicate significant differences at (p ≤ 0.05, 0.01, and 0.001, respectively; ns: not significant).

Table S9. Concentration and content of flavonols in 2010/2011(Blancquaert, 2015).

Concentration (mg/g skin)						Total flavonol content (mg/berry)
**DAA**	**Treatment**	**Quercetin-rutinoside**	**Quercetin-galactoside**	**Quercetin-glucoside**	**Quercetin-glucuronide**	
13	Standard (Control)	0.01 ± 0	0.00 ± 0	0.01 ± 0	0.16 ± 0.03 b	0.011 ± 0.004 a
	Leaf Removal West	0.02 ± 0.01	0.01 ± 0.01	0.03 ± 0.01	0.28 ± 0.08 a	0.0003 ± 0 b
	STD-UV-B	0.01 ± 0.01	0.01 ± 0.01	0.03 ± 0.03	0.21 ± 0.10 ab	0.014 ± 0.005 a
	LRW-UV-B	0.01 ± 0	0.00 ± 0	0.01 ± 0	0.16 ± 0.03 b	0.0003 ± 0 b
Significance		***ns***	*ns*	*ns*	*	***
17	Standard (Control)	0.01 ± 0.01	0.01 ± 0	0.01 ± 0.01	0.23 ± 0.06	0.015 ± 0.004 a
	Leaf Removal West	0.03 ± 0.02	0.01 ± 0.01	0.05 ± 0.05	0.33 ± 0.25	0.0± 0 b
	STD-UV-B	0.02 ± 0	0.01 ± 0	0.03 ± 0.01	0.25 ± 0.05	0.017 ± 0.005 a
	LRW-UV-B	0.01 ± 0.01	0.01 ± 0	0.01 ± 0.01	0.23 ± 0.06	0.0000 ± 0 b
Significance		*ns*	*ns*	*ns*	*ns*	***
22	Standard (Control)	0.01 ± 0 b	0.00 ± 0 b	0.01 ± 0 b	0.15 ± 0.03 b	0.010 ± 0.002 a
	Leaf Removal West	0.03 ± 0.02 a	0.01 ± 0.01 a	0.06 ± 0.05 a	0.38 ± 0.16 a	0 ± 0 b
	STD-UV-B	0.01 ± 0.01 b	0.00 ± 0 b	0.02 ± 0.01 b	0.14 ± 0.07 b	0 ± 0 b
	LRW-UV-B	0.01 ± 0 b	0.00 ± 0 b	0.01 ± 0 b	0.15 ± 0.03 b	0.010 ± 0.006 a
Significance		**	**	**	***	***
48	Standard (Control)	0.03 ± 0.01 b	0.01 ± 0 b	0.05 ± 0.02	0.35 ± 0.08 b	0.038 ± 0.011 a
	Leaf Removal West	0.05 ± 0.01 a	0.03 ± 0 a	0.12 ± 0.03	0.54 ± 0.13 a	0.0 ± 0 c
	STD-UV-B	0.01 ± 0.01 c	0.00 ± 0 c	0.02 ± 0.01	0.16 ± 0.05 c	0.016 ± 0 b
	LRW-UV-B	0.01 ± 0 c	0.01 ± 0 c	0.03 ± 0.01	0.15 ± 0.04 bc	0.0 ± 0.006 c
Significance		*******	*******	***	***	***
62	Standard (Control)	0.02 ± 0.01 b	0.04 ± 0.02 a	0.25 ± 0.07 b	0.34 ± 0.07 b	0.055 ± 0.019 a
	Leaf Removal West	0.04 ± 0.01 a	0.06 ± 0.02 a	0.36 ± 0.14 a	0.63 ± 0.19 a	0 ± 0 c

	STD-UV-B	0.01 ± 0.01 b	0.00 ± 0 b	0.07 ± 0.02 c	0.15 ± 0.05 c	0.024 ± 0.007 b
	LRW-UV-B	0.01 ± 0 b	0.01 ± 0.01 b	0.08 ± 0.03 c	0.15 ± 0.05 c	0 ± 0 c
Significance		***	***	***	***	***
76	Standard (Control)	0.01 ± 0 b	0.04 ± 0 b	0.26 ± 0.02 b	0.32 ± 0.03 b	0.083 ± 0.021 a
	Leaf Removal West	0.04 ± 0.02 a	0.09 ± 0.02 a	0.52 ± 0.11 a	0.59 ± 0.11 a	0 ± 0 c
	STD-UV-B	0.01 ± 0 b	0.00 ± 0.01 c	0.09 ± 0.01 c	0.19 ± 0.04 c	0.034 ± 0.004 b
	LRW-UV-B	0.01 ± 0 b	0.00 ± 0 c	0.10 ± 0.03 c	0.20 ± 0.07 c	0 ± 0 c
Significance		***	***	***	***	***
90	Standard (Control)	0.01 ± 0 b	0.04 ± 0	0.26 ± 0.02 b	0.32 ± 0.03 b	0.085 ± 0.005 a
	Leaf Removal West	0.03 ± 0.01 a	0.09 ± 0.03	0.51 ± 0.21 a	0.56 ± 0.15 a	0.00 ± 0 c
	STD-UV-B	0.01 ± 0.01 b	0.00 ± 0	0.08 ± 0.02 c	0.24 ± 0.08 bc	0.043 ± 0.012 b
	LRW-UV-B	0.01 ± 0.01 b	0.00 ± 0	0.07 ± 0.02 c	0.18 ± 0.05 c	0.00 ± 0 c
Significance		**	***	***	***	
116	Standard (Control)	0.01 ± 0.01 b	0.04 ± 0.02 b	0.24 ± 0.09 b	0.28 ± 0.07 b	0.08 ± 0.02 a
	Leaf Removal West	0.02 ± 0 a	0.10 ± 0.02a	0.63 ± 0.14 a	0.49 ± 0.06 a	0.0 ± 0 c
	STD-UV-B	0.00 ± 0 c	0.00 ± 0 c	0.05 ± 0.01 c	0.13 ± 0.04 c	0.024 ± 0.005 b
	LRW-UV-B	0.01 ± 0 c	0.00 ± 0 c	0.06 ± 0.02 c	0.15 ± 0.02 c	0.0 ± 0 c
Significance		***	***	***	***	***

Each value represents the mean of 5 replicates ± standard deviation in concentration (mg/g skin) and content (mg/berry). Treatments: STD (Shaded/Control); LRW (Leaf Removal West); STD-UV-B (STD with decreased UV-B radiation); LRW-UV-B (LRW with decreased UV-B radiation). Different letters indicate significant differences at ($p \leq 0.05$, 0.01, and 0.001, respectively; ns: not significant).

Table S10. Concentration and content of flavonols in 2011/2012 (Blancquaert, 2015).

Concentration (mg/g skin)						Total flavonol content (mg/berry)
**DAA**	**Treatment**	**Quercetin-rutinoside**	**Quercetin-galactoside**	**Quercetin-glucoside**	**Quercetin-glucuronide**	
26	Standard (Control)	0.01 ± 0.01 b	0.01 ± 0 b	0.02 ± 0.01 b	0.20 ± 0.08 b	0.015 ± 0.008 b
	Leaf Removal West	0.03 ± 0.01 a	0.01 ± 0 a	0.04 ± 0.01 a	0.34 ± 0.06 a	0.027 ± 0.006 a
	LR (-UV-B, 2xOp50)	0.00 ± 0 b	0.002 ± 0.001 c	0.01 ± 0 b	0.12 ± 0.01 c	0.009 ± 0.002 b
	LR(-UV-B, 2xUHI)	0.01 ± 0 b	0.003 ± 0.002 bc	0.01 ± 0.01 b	0.14 ± 0.04bc	0.009 ± 0.003 b
Significance		*******	***	***	***	***
33	Standard (Control)	0.02 ± 0.01 b	0.01 ± 0 b	0.03 ± 0.01 b	0.24 ± 0.05 b	0.020 ± 0.005 b
	Leaf Removal West	0.06 ± 0.02 a	0.03 ± 0.01 a	0.07 ± 0.02 a	0.55 ± 0.12 a	0.053 ± 0.012 a
	LR (-UV-B, 2xOp50)	0.01 ± 0 c	0.003 ± 0.001 b	0.02 ± 0.01 b	0.13 ± 0.05 c	0.010 ± 0.004 b
	LR(-UV-B, 2xUHI)	0.01 ± 0 c	0.003 ± 0.001 b	0.02 ± 0 b	0.13 ± 0.04 c	0.012 ± 0.004 b
Significance		***	***	***	***	***
40	Standard (Control)	0.05 ± 0.02 b	0.02 ± 0.01 b	0.06 ± 0.02 b	0.39 ± 0.13 b	0.039 ± 0.015 b
	Leaf Removal West	0.10 ± 0.01 a	0.04 ± 0 a	0.12 ± 0.01 a	0.70 ± 0.07 a	0.071 ± 0.007 a
	LR (-UV-B, 2xOp50)	0.01 ± 0.01 c	0.01 ± 0 c	0.02 ± 0.01 c	0.16 ± 0.06 c	0.015 ± 0.005 c
	LR(-UV-B, 2xUHI)	0.01 ± 0.01 c	0.004 ± 0 c	0.02 ± 0.01 c	0.15 ± 0.04 c	0.014 ± 0.004 c
Significance		***	***	***	***	***
47	Standard (Control)	0.04 ± 0.01 b	0.02 ± 0 b	0.06 ± 0.01 b	0.36 ± 0.06 b	0.036 ± 0.005 b
	Leaf Removal West	0.11 ± 0.01 a	0.04 ± 0 a	0.15 ± 0.04 a	0.87 ± 0.11 a	0.084 ± 0.008 a
	LR (-UV-B, 2xOp50)	0.01 ± 0 c	0.003 ± 0 c	0.01 ± 0 c	0.11 ± 0.02 c	0.010 ± 0.001 c
	LR(-UV-B, 2xUHI)	0.01 ± 0 c	0.004 ± 0 c	0.02 ± 0.01 c	0.14 ± 0.03 c	0.012 ± 0.002 c
Significance		*******	*******	***	***	***
54	Standard (Control)	0.06 ± 0.02 b	0.03 ± 0.01 b	0.11 ± 0.03 b	0.55 ± 0.17 b	0.052 ± 0.018 b
	Leaf Removal West	0.12 ± 0.01 a	0.06 ± 0 a	0.17 ± 0.04 a	0.96 ± 0.07 a	0.099 ± 0.008 a

	LR (-UV-B, 2xOp50)	0.01 ± 0.01 c	0.01 ± 0 c	0.02 ± 0.01 c	0.18 ± 0.10 c	0.017 ± 0.009 c
	LR(-UV-B, 2xUHI)	0.01 ± 0 c	0.01 ± 0 c	0.03 ± 0 c	0.16 ± 0.02 c	0.015 ± 0.001 c
Significance		***	***	***	***	***
68	Standard (Control)	0.03 ± 0.01 b	0.05 ± 0.01 b	0.21 ± 0.06 b	0.47 ± 0.11 b	0.086 ± 0.042 b
	Leaf Removal West	0.08 ± 0.01 a	0.09 ± 0.03 a	0.41 ± 0.12 a	0.94 ± 0.13 a	0.212 ± 0.037 a
	LR (-UV-B, 2xOp50)	0.01 ± 0.01 c	0.02 ± 0.01 c	0.10 ± 0.07bc	0.28 ± 0.15 c	0.081 ± 0.036 b
	LR(-UV-B, 2xUHI)	0.02 ± 0 c	0.02 ± 0.01 c	0.04 ± 0.05 c	0.10 ± 0.06 d	0.082 ± 0.024 b
Significance		***	***	***	***	***
82	Standard (Control)	0.03 ± 0.01 b	0.06 ± 0.03 b	0.21 ± 0.13 b	0.50 ± 0.23 b	0.107 ± 0.041 b
	Leaf Removal West	0.09 ± 0.03 a	0.15 ± 0.02 a	0.56 ± 0.21 a	1.25 ± 0.46 a	0.20 ± 0.035 a
	LR (-UV-B, 2xOp50)	0.01 ± 0.01 b	0.05 ± 0.04 b	0.19 ± 0.11 b	0.41 ± 0.13 b	0.058 ± 0.027 c
	LR(-UV-B, 2xUHI)	0.02 ± 0.01 b	0.05 ± 0.02 b	0.24 ± 0.07 b b	0.46 ± 0.07	0.030 ± 0.010 c
Significanc*e*		***	***	***	***	***
96	Standard (Control)	0.02 ± 0.01 b	0.07 ± 0.03 b	0.36 ± 0.16 b	0.33 ± 0.11 b	0.107 ± 0.041 b
	Leaf Removal West	0.04 ± 0 a	0.14 ± 0.03 a	0.68 ± 0.15 a	0.68 ± 0.06 a	0.200 ± 0.035 a
	LR (-UV-B, 2xOp50)	0.01 ± 0 c	0.04 ± 0.01 c	0.15 ± 0.10 c	0.27 ± 0.12bc	0.058 ± 0.027
	LR(-UV-B, 2xUHI)	0.01 ± 0 c	0.02 ± 0.01 c	0.09 ± 0.06 c	0.17 ± 0.06 c	0.030 ± 0.010 c
Significance		***	***	***	***	***
110	Standard (Control)	0.01 ± 0 b	0.06 ± 0.02 b	0.30 ± 0.09 b	0.31 ± 0.03bc	0.084 ± 0.019 b
	Leaf Removal West	0.04 ± 0.01 a	0.13 ± 0.05 a	0.73 ± 0.14 a	0.63 ± 0.12a	0.205 ± 0.042 a
	LR (-UV-B, 2xOp50)	0.01 ± 0 b	0.05 ± 0.01 b	0.25 ± 0.05 b	0.40 ± 0.06 b	0.083 ± 0.016 b
	LR(-UV-B, 2xUHI)	0.01 ± 0 b	0.005 ± 0 c	0.18 ± 0.14 b	0.30 ± 0.06 c	0.054 ± 0.023 b
Significance		***	***	***	***	***
130	Standard (Control)	0.01 ± 0.01 b	0.04 ± 0.01 b	0.29 ± 0.09 b	0.27 ± 0.06 b	0.080 ± 0.020 b
	Leaf Removal West	0.02 ± 0 a	0.09 ± 0.02 a	0.52 ± 0.06 a	0.40 ± 0.07 a	0.149 ± 0.017 a

	LR (-UV-B, 2xOp50)	0.007 ± 0bc	0.03 ± 0.01 b	0.17 ± 0.07 c	0.20 ± 0.05bc	0.054 ± 0.021 c
	LR(-UV-B, 2xUHI)	o.oo5 ± 0 c	0.00 ± 0 c	0.11 ± 0.05 c	0.17 ± 0.04 c	0.038 ± 0.009 c
Significance		***	*******	***	***	***

Each value represents the mean of 5 replicates (±) standard deviation in concentration (mg/g skin) and content (mg/berry).Treatments: STD (Shaded/Control); LRW (Leaf Removal West); LR-UV-B, 2xOp50 (Leaf removal with decreased UV-B radiation and 2xOp50 UV-sheets added on both sides of the bunch zone); LR-UV-B, 2xUHI (Leaf removal with decreased UV-B radiation and 2xUHI UV-sheets added on both sides of the bunch zone). Different letters indicate significant differences at ($p \leq 0.05$, 0.01, and 0.001, respectively; ns: not significant).
